# Maternal immune activation induces sustained changes in fetal microglia motility

**DOI:** 10.1038/s41598-020-78294-2

**Published:** 2020-12-07

**Authors:** Kana Ozaki, Daisuke Kato, Ako Ikegami, Akari Hashimoto, Shouta Sugio, Zhongtian Guo, Midori Shibushita, Tsuyako Tatematsu, Koichiro Haruwaka, Andrew J. Moorhouse, Hideto Yamada, Hiroaki Wake

**Affiliations:** 1 Division of System Neuroscience, Kobe University Graduate School of Medicine, Kobe, Japan; 2 Department of Obstetrics and Gynecology, Kobe University Graduate School of Medicine, Kobe, Japan; 3 Department of Anatomy and Molecular Cell Biology, Nagoya University Graduate School of Medicine, Nagoya, Japan; 4 School of Medical Sciences, The University of New South Wales, Sydney, Australia; 5 Core Research for Evolutional Science and Technology, Japan Science and Technology Agency, Saitama, Japan

**Keywords:** Development of the nervous system, Glial development, Neuroscience, Diseases of the nervous system, Autism spectrum disorders

## Abstract

Maternal infection or inflammation causes abnormalities in brain development associated with subsequent cognitive impairment and in an increased susceptibility to schizophrenia and autism spectrum disorders. Maternal immune activation (MIA) and increases in serum cytokine levels mediates this association via effects on the fetal brain, and microglia can respond to maternal immune status, but consensus on how microglia may respond is lacking and no-one has yet examined if microglial process motility is impaired. In this study we investigated how MIA induced at two different gestational ages affected microglial properties at different developmental stages. Immune activation in mid-pregnancy increased IL-6 expression in embryonic microglia, but failed to cause any marked changes in morphology either at E18 or postnatally. In contrast MIA, particularly when induced earlier (at E12), caused sustained alterations in the patterns of microglial process motility and behavioral deficits. Our research has identified an important microglial property that is altered by MIA and which may contribute to the underlying pathophysiological mechanisms linking maternal immune status to subsequent risks for cognitive disease.

## Introduction

Infection with influenza virus, Toxoplasma gondii, rubella virus, cytomegalovirus or herpes simplex virus type 2 during pregnancy has been associated with a higher incidence of schizophrenia and autism spectrum disorders in offspring^1–10^. Maternal inflammation leads to elevated pro-inflammatory cytokines such as IFN gamma (IFNγ) and interleukins (IL) 4, 5 and 6, and these are believed to mediate the effects of infection on fetal brain development and subsequent cognitive disease outcomes^11^. For example, administration of IL-6 to pregnant mice at 12 days gestation (E12) replicates the effects of maternal infection, resulting in altered gene expression in the fetal cortex and subsequent behaviors in the offspring characteristic of schizophrenia and ASD, including abnormalities in pre-pulse inhibition (PPI), latent inhibition (LI), social interactions, and open field behaviours^12^. Antibodies to IL-6 administered to pregnant dams, or genetic deletion of IL-6, can reduce these subsequent behavioral abnormalities whether induced by either gestational IL-6 injection, or by maternal Poly(I:C)-induced infection^12^. Hence, there is a strong case for MIA contributing to cognitive symptoms in offspring in both rodents and humans.

Microglia have been implicated in these links between maternal inflammation and the offspring’s brain development. These immune-surveillant cells in the central nervous system (CNS) contribute to sculpting neural circuits during pre- and postnatal development through regulating neuron apoptosis and neurogenesis, and via phagocytosis and formation of synapses^13,14^. Microglia are readily transformed into a reactive state by peripheral or central inflammation^15^, and MIA can also induce an altered microglial phenotype in the postnatal and adult brain of offspring in some studies. However, these results have been quite variable, depending in part on the age examined and specific parameter measured, and the way in which MIA is induced^16^. Their acute response to MIA in the fetal brains is also variable, with further complications arising from the normal morphological and biochemical changes in microglia during fetal development^16,17^. Despite these challenges, it is important to track how microglia respond across development to MIA, as targeting specific changes in microglia holds promise for reducing cognitive effects of schizophrenia and potentially other MIA-related diseases^18,19^. Therefore, in this study, we induced MIA by Poly(I:C) injection at two different gestational days (E12 and E15) and characterized the biochemical and morphological microglial phenotypes at different developmental stages in offspring, from fetal to young adult. Furthermore, we used two-photon imaging to quantify microglial process motility, as we were unaware of any prior studies that examined how MIA effects this parameter. Microglial process motility is a key physiological parameter linked to their immune-surveillant and neuronal homeostasis functions, and we hypothesized that MIA may alter this motility. We found that maternal inflammation caused by E12 and E15 Poly(I:C) -injection consistently lead to increased IL-6 expression in liver, placenta and in fetal microglia. Microglial morphological parameters were unchanged in fetal and postnatal brains by E12 and E15 Poly(I:C) -injection, with only subtle changes in other biochemical phenotypes. However, MIA induced changes in process motility in embryonic microglia, and changes persisted into the postnatal and adolescent stages, albeit in different directions. Our results add an important functional parameter to the range of persistent changes seen in microglia following MIA that adds support to microglial abnormalities contributing to the link between maternal inflammation and subsequent cognitive disorders in offspring.

## Results

### Pattern of maternal and fetal cytokine changes following Poly(I:C) injection at E12 or E15

**Figure 1:**
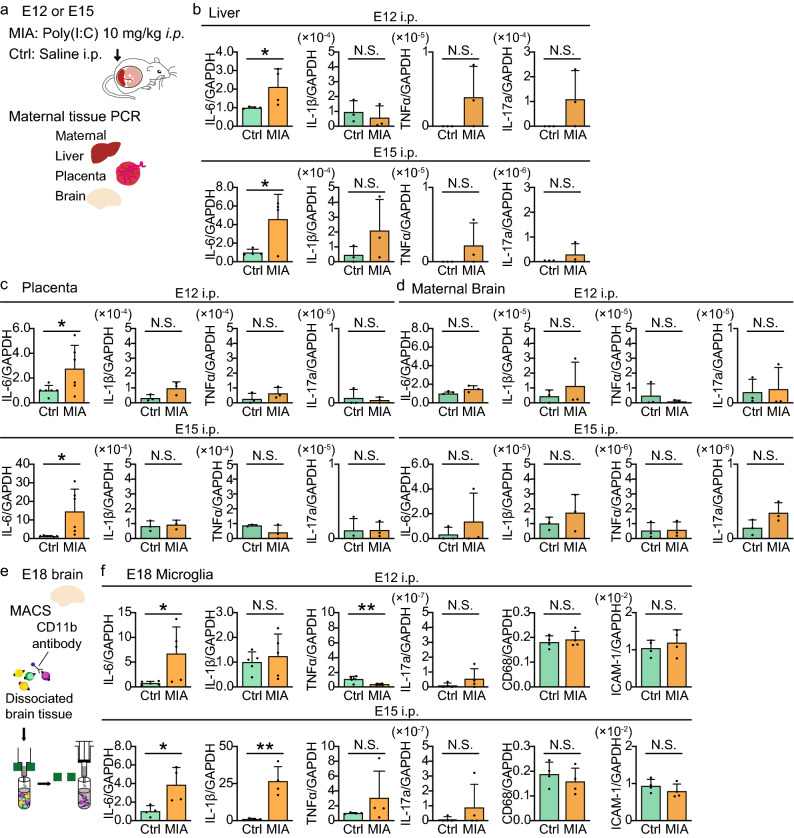
Maternal immune activation at E12 and E15 causes an altered biochemical phenotype in E18 fetal microglia. (**a**) Experimental scheme showing MIA induced in mothers by Poly(I:C) injection (*i.p.*) at gestational day 12 or 15 (E12, E15) and subsequent maternal tissue extraction. (**b**–**d**) Relative cytokine mRNA expression levels in maternal liver (IL-6: *n* = 4 mice in each group; IL-1β, TNFα and IL-17a: *n* = 3 mice in each group), placenta (IL-6: *n* = 6 mice in each group; IL-1β, TNFα and IL-17a: *n* = 3 mice in each group) and brain (*n* = 3 mice in each group). Control (Ctrl: saline -injected) versus MIA (E12 and E15 Poly(I:C) -injected) mice. (**e**) Experimental schema showing extraction of microglia (CD11b positive cells) from fetal brain. (**f**) Relative cytokine mRNA expression levels in E18 microglia (IL-6, IL-1β and TNFα: *n* = pooled embryos from 5 (E12) or 4 (E15) mice; IL-17a, CD68 and ICAM-1: *n* = pooled embryos from 2 mice in each group). In (**b**–**d**, **f**) Ctrl represents age-matched saline injection and MIA is E12 or E15 Poly(I:C) -injected mice. Graphs represent the mean ± standard deviation, while dots represent data from each mouse. *P < 0.05, **P < 0.01 and N.S.: not significant, unpaired *t*-test.

To induce MIA, we injected intraperitoneal Poly(I:C) to dams on two gestational days, E12 or E15 (birth is typically at E21). Poly(I:C) is a synthetic analogue which mimics the viral double-stranded RNA that activates Toll-like receptor 3, the innate immune receptor which promotes antiviral innate immune response^20,21^. Control mice were injected with saline and the mRNA expression profile of pro-inflammatory molecules was measured by real time PCR (Fig. 1), in fetal microglia, and in the maternal liver, placenta and brain. IL-6, IL-1β, TNFα and IL-17a, and the cell surface markers CD68 and ICAM-1, have all been previously implicated as key pro-inflammatory molecules that may mediate the behavioral consequences of maternal infection in the offspring^22^. Injections at both gestational day E12 (E12 Poly(I:C) -injection) and E15 (E15 Poly(I:C) -injection) significantly increased expression of IL-6 in both the maternal liver and placenta, but not in the maternal brain (Fig. 1b–d and Table 1). The levels of all the other cytokines (IL-1β, TNFα, IL-17a) did not change in maternal tissues from E12 or E15 MIA. (Fig. 1b–d, Table 1). CD68 expression was significantly increased in the maternal brain following E15 Poly(I:C) -injection, while no difference was seen for ICAM-1 expression (supplementary Fig. S1, Table 1). To investigate how MIA influenced expression of these pro-inflammatory molecules in the fetal brain, we isolated microglia from E18 fetal cortices using magnetic-activated cell sorting (MACS) system with CD11b magnetic beads (Fig. 1e). Increased IL-6 expression was also observed in fetal microglia, after both E12 and E15 Poly(I:C) -injection (Fig. 1f). The cytokine profile depended on the timing of MIA: IL-1β increased in E15 Poly(I:C) -injected mice but not in E12 Poly(I:C) -injected mice, while TNFα was decreased in E12 Poly(I:C) -injected mice but was unchanged in E15 Poly(I:C) -injected mice. We did not detect any differences in the expression of IL-17a, CD68 and ICAM-1 (Fig. 1f and Table 1).

**Table 1:** Cytokines analysis of maternal organs and E18 microglia.

	E12 Ctrl	E12 MIA	*P*	*N*	E15 Ctrl	E15 MIA	*P*	*N*
**Liver**								
IL-6	1.0 ± 0.045	2.3 ± 0.82	0.021	4 vs. 4	1.0 ± 0.35	4.6 ± 2.7	0.038	4 vs. 4
IL-1β	0.97 × 10^−4^ ± 0.75 × 10^−4^	0.55 × 10^−4^ ± 0.79 × 10^−4^	0.54	3 vs. 3	0.46 × 10^−4^ ± 0.56 × 10^−4^	2.0 × 10^−4^ ± 2.0 × 10^−4^	0.26	3 vs. 3
TNFα	0.0 ± 0.0	0.39 × 10^−5^ ± 0.42 × 10^−5^	0.18	3 vs. 3	0.0 ± 0.0	0.22 × 10^−5^ ± 0.33 × 10^−5^	0.28	3 vs. 3
IL-17a	0.0 ± 0.0	1.10 × 10^−4^ ± 1.2 × 10^−4^	0.18	3 vs. 3	0.0 ± 0.0	0.30 × 10^−6^ ± 0.43 × 10^−6^	0.30	3 vs. 3
CD68	0.23 × 10^−3^ ± 0.13 × 10^−3^	0.16 × 10^−3^ ± 0.15 × 10^−3^	0.56	3 vs. 3	0.081 × 10^−3^ ± 0.14 × 10^−3^	0.64 × 10^−3^ ± 0.59 × 10^−3^	0.19	3 vs. 3
ICAM-1	0.35 × 10^−2^ ± 0.12 × 10^−2^	0.33 × 10^−2^ ± 0.13 × 10^−2^	0.85	3 vs. 3	0.34 × 10^−2^ ± 0.49 × 10^−3^	0.61 × 10^−2^ ± 0.25 × 10^−2^	0.14	3 vs. 3
**Placenta**								
IL-6	1.0 ± 0.40	2.8 ± 1.9	0.049	6 vs. 6	1.0 ± 0.28	14.6 ± 12.0	0.019	6 vs. 6
IL-1β	0.33 × 10^−4^ ± 0.22 × 10^−4^	0.99 × 10^−4^ ± 0.42 × 10^−4^	0.075	3 vs. 3	0.84 × 10^−4^ ± 0.35 × 10^−4^	0.94 × 10^−4^ ± 0.30 × 10^−4^	0.72	3 vs. 3
TNFα	0.31 × 10^−4^ ± 0.41 × 10^−4^	0.68 × 10^−4^ ± 0.43 × 10^−4^	0.34	3 vs. 3	0.88 × 10^−4^ ± 0.57 × 10^−5^	0.42 × 10^−4^ ± 0.48 × 10^−4^	0.17	3 vs. 3
IL-17a	0.70 × 10^−6^ ± 1.0 × 10^−6^	0.38 × 10^−6^ ± 0.39 × 10^−6^	0.70	3 vs. 3	1.1 × 10^−6^ ± 1.6 × 10^−6^	1.2 × 10^−6^ ± 1.1 × 10^−6^	0.97	3 vs. 3
CD68	0.20 ± 0.085	0.22 ± 0.050	0.70	3 vs. 3	0.16 ± 0.14	0.17 ± 0.060	0.90	3 vs. 3
ICAM-1	0.59 × 10^−3^ ± 0.50 × 10^−3^	1.08 × 10^−3^ ± 0.27 × 10^−3^	0.21	3 vs. 3	0.84 × 10^−3^ ± 0.76 × 10^−3^	1.5 × 10^−3^ ± 0.44 × 10^−3^	0.29	3 vs. 3
**Brain**								
IL-6	1.0 ± 0.16	1.5 ± 0.35	0.090	3 vs. 3	0.33 ± 0.58	1.37 ± 2.3	0.49	3 vs. 3
IL-1β	0.45 × 10^−5^ ± 0.42 × 10^−5^	1.1 × 10^−5^ ± 1.6 × 10^−5^	0.51	3 vs. 3	1.0 × 10^−5^ ± 0.42 × 10^−5^	1.7 × 10^−5^ ± 1.2 × 10^−5^	0.39	3 vs. 3
TNFα	0.49 × 10^−5^ ± 0.79 × 10^−5^	0.97 × 10^−6^ ± 0.84 × 10^−6^	0.44	3 vs. 3	0.54 × 10^−6^ ± 0.53 × 10^−6^	0.59 × 10^−6^ ± 0.53 × 10^−6^	0.91	3 vs. 3
IL-17a	0.15 × 10^−5^ ± 0.17 × 10^−5^	0.18 × 10^−5^ ± 0.30 × 10^−5^	0.85	3 vs. 3	0.15 × 10^−6^ ± 0.10 × 10^−6^	0.35 × 10^−6^ ± 0.13 × 10^−6^	0.11	3 vs. 3
CD68	5.2 × 10^−3^ ± 0.73 × 10^−3^	5.6 × 10^−3^ ± 0.46 × 10^−3^	0.48	3 vs. 3	5.5 × 10^−3^ ± 0.54 × 10^−3^	7.0 × 10^−3^ ± 0.29 × 10^−3^	0.016	3 vs. 3
ICAM-1	2.3 × 10^−4^ ± 0.34 × 10^−4^	1.9 × 10^−4^ ± 0.70 × 10^−4^	0.49	3 vs. 3	3.3 × 10^−4^ ± 1.1 × 10^−4^	3.6 × 10^−4^ ± 0.68 × 10^−4^	0.73	3 vs. 3
**E18 microglia**								
IL-6	1.0 ± 0.44	8.9 ± 6.5	0.040	5 vs. 5 (5 pooled embryos from 5 mice)	1.0 ± 0.61	3.6 ± 1.9	0.028	4 vs. 4 (4 pooled embryos from 4 mice)
IL-1β	1.0 ± 0.42	1.2 ± 0.90	0.60	5 vs. 5 (5 pooled embryos from 5 mice)	1.0 ± 0.57	26 ± 8.5	0.0021	4 vs. 4 (4 pooled embryos from 4 mice)
TNFα	1.0 ± 0.38	0.31 ± 0.11	0.0078	5 vs. 5 (5 pooled embryos from 5 mice)	1.0 ± 0.10	3.1 ± 3.6	0.30	4 vs. 4 (4 pooled embryos from 4 mice)
IL-17a	0.10 × 10^−7^ ± 0.16 × 10^−7^	0.56 × 10^−7^ ± 0.71 × 10^−7^	0.26	4 vs. 4 (4 pooled embryos from 2 mice)	0.090 × 10^−7^ ± 0.18 × 10^−7^	0.90 × 10^−7^ ± 1.6 × 10^−7^	0.34	4 vs. 4 (4 pooled embryos from 2 mice)
CD68	0.18 ± 0.025	0.19 ± 0.033	0.62	4 vs. 4 (4 pooled embryos from 2 mice)	0.19 ± 0.048	0.16 ± 0.054	0.44	4 vs. 4 (4 pooled embryos from 2 mice)
ICAM-1	1.1 × 10^−2^ ± 0.21 × 10^−2^	1.2 × 10^−2^ ± 0.34 × 10^−2^	0.48	4 vs. 4 (4 pooled embryos from 2 mice)	0.94 × 10^−2^ ± 0.17 × 10^−2^	0.80 × 10^−2^ ± 0.19 × 10^−2^	0.31	4 vs. 4 (4 pooled embryos from 2 mice)

### Effects of maternal inflammation on microglial morphology

Microglia recognized to exist in different functional phenotypes that include surveillant modes in healthy brain, and reactive phenotypes in the inflamed/injured brain, with these phenotypes having different biochemical and morphological characteristics^23^. The different microglial cytokine expression patterns observed above are consistent with MIA inducing a change in microglial phenotype, as observed previously^16^.

**Figure 2:**
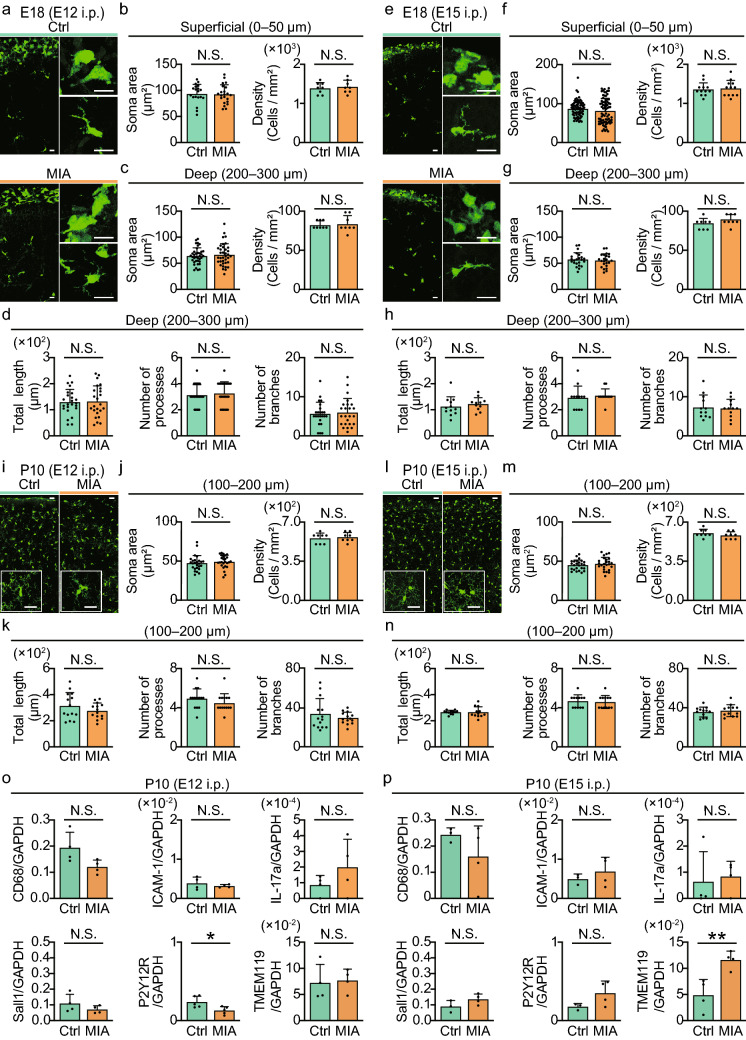
Postnatal (P10) microglia morphology is unchanged by MIA. (**a**) Typical images of microglia (CX_3_CR1-+’ve cells) in the somatosensory cortex of the E18 fetal brain isolated from prior E12 saline (Ctrl, upper) or Poly(I:C) (MIA, lower) -injected mice. Representative magnified images on the right. Scale bars, 20 μm. (**b**) Corresponding quantification of the soma area and density of microglia in superficial cortex (soma area; Ctrl: *n* = 19 cells from 4 mice; MIA: *n* = 22 cells from 4 mice; cell density; *n* = 8 fields from 4 mice in each group). (**c**, **d**) Quantification of the soma area and density (**c**), or process complexity (**d**) of E18 microglia in deeper cortical layers in E12 Ctrl and MIA mice (soma area; *n* = 37 cells from 4 mice in each group; cell density; *n* = 8 fields from 4 mice in each group; total process length, number of processes and number of process branches; *n* = 24 cells from 4 mice in each group). (**e**–**h**) As in (**a**–**d**), but for E18 microglia from E15 saline (Ctrl) or Poly(I:C) (MIA) -injected mice, showing representative images (**e**) scale bars, 20 μm. (**f**) Soma area and density in superficial cortical layer (soma area; Ctrl: *n* = 67 cells from 5 mice; MIA: *n* = 75 cells from 5 mice; cell density; *n* = 12 fields from 5 mice in each group), (**g**, **h**) soma area, density, and process parameters for microglia in deeper cortical layers (soma area; *n* = 23 cells from 4 mice in each group; cell density; *n* = 8 fields from 4 mice in each group, total process length, number of processes and number of process branches; *n* = 12 cells from 4 mice in each group). (**i**–**k**) Typical images (**i**), microglia soma area and density (**j**) and microglia process parameters (**k**) for microglia imaged from the somatosensory cortex of P10 offspring of E12 saline (Ctrl) or Poly(I:C) (MIA) -injected mice. Scale bars in (**i**), 20 μm, soma area in (**j**): (*n* = 26 cells from 4 mice in each group; cell density in (**j**): *n* = 8 fields from 4 mice in each group; (**k**): total process length, number of processes and number of process branches; *n* = 14 cells from 4 mice in each group. (**l**–**n**) As in (**i**–**k**) but for microglia in P10 offspring of E15 saline (Ctrl) or Poly(I:C) (MIA) -injected mice. Scale bars in (**l**), 20 μm, (**m**, **n**): (soma area; *n* = 25 cells from 4 mice in each group; cell density; *n* = 8 fields from 4 mice in each group; total process length, number of processes and number of process branches; *n* = 12 cells from 4 mice in each group). (**o**, **p**) Comparison of the mRNA expression levels of specific microglia phenotypic markers in microglia isolated from brains of P10 offspring from E12 (**o**) or E15 (**p**) saline (Ctrl) or Poly (I:C) (MIA) -injected mice. P2Y12R expression was significantly reduced in E12 MIA mice (*n* = 5 mice in each group) while TMEM119 expression was increased in E15 MIA mice (*n* = 4 mice in each group). No difference in expression of CD68, ICAM-1 IL-17a and Sall1 were seen between Ctrl and MIA groups. In all graphs columns and error bars represent the mean ± standard deviation while dots represent individual experiments. *P < 0.05, **P < 0.01 and N.S.: not significant, unpaired *t*-test.

Therefore, we next examined if MIA also altered microglial morphology. We used fetal brains from CX_3_CR1-EGFP mice so as to visualize CX_3_CR1-+’ve cells with EGFP fluorescence, and injected dams with either saline or Poly(I:C) at E12 or E15. Fetal brains at E18 were fixed and microglia in cortical layers were quantified in coronal brain slices. As peripheral macrophages populate the brain surface^24^, we distinguished fluorescent cells in the dense superficial (0–50 μm), and more sparse deeper (200–300 μm) layers, and quantified soma areas, cell density, total process length, number of processes, and number of process branches—all typical morphological parameters that can change when adult microglia are activated (Fig. 2a–h)^25–27^. None of these parameters changed for neither the E12 or the E15 Poly(I:C) -injected mice (Fig. 2a–h and Table 2) as compared to control mice, for either superficial or deeper layers. We also similarly examined morphological parameters in microglia from brains of mice at 10 days postnatal (P10) (located at 100–200 μm below from the surface), following E12 and E15 Poly(I:C) -injection. Although the absolute microglial density increased with maturation, again there were no differences in any morphological parameters compared to age-matched control mice (Fig. 2i–n). Finally, we isolated microglia from P10 cortices using MACS and examined gene expression profiles following E12 or E15 Poly(I:C) -injection. We focused on microglia-specific genes related to inflammation^28–31^ and differentiation^32^ (Fig. 2o, p and Table 3). There were no differences in the gene expression levels of CD68, ICAM-1, IL-17a or Sall1 following either E12 or E15 Poly(I:C) -injection. In contrast, TMEM119 expression was selectively increased in P10 microglia from E15 Poly(I:C) -injected mice whereas P2Y12R expression was significantly reduced in E12 Poly(I:C) -injected mice (Fig. 2o, p and Table 3). These results suggest that MIA is not associated with microglial morphological changes but can cause some subtle differences in the level of postnatal microglial differentiation, with this difference depending on the timing of MIA.

**Table 2:** Morphological analysis of microglia at E18 and P10.

E18						
Mouse, *P* value	E12 Ctrl	E12 MIA	*P* value	E15 Ctrl	E15 MIA	*P* value
**Soma area (0–50 μm)**						
Mean ± SD (μm^2^)	93.2 ± 18.4	92.4 ± 18.2	0.89	85.0 ± 20.6	79.7 ± 31.7	0.74
Number of cells	19 (from 4 mice)	22 (from 4 mice)		67 (from 5 mice)	75 (from 5 mice)	
**Density (0–50 μm)**						
Mean ± SD (cells/mm^2^)	1388 ± 146	1425 ± 167	0.24	1350 ± 168	1375 ± 205	0.75
Number of fields	8 (from 4 mice)	8 (from 4 mice)		12 (from 5 mice)	12 (from 5 mice)	
**Soma area (200–300 μm)**						
Mean ± SD (μm^2^)	63.4 ± 15.5	65.7 ± 21.1	0.59	56.6 ± 13.0	54.8 ± 12.8	0.63
Number of cells	37 (from 4 mice)	37 (from 4 mice)		23 (from 4 mice)	23 (from 4 mice)	
**Density (200–300 μm)**						
Mean ± SD (cells/mm^2^)	81.7 ± 5.05	83.0 ± 10.4	0.77	85.4 ± 6.90	90.3 ± 6.90	0.18
Number of fields	8 (from 4 mice)	8 (from 4 mice)		8 (from 4 mice)	8 (from 4 mice)	
**Total process length (200–300 μm)**						
Mean ± SD (μm)	129 ± 48.9	132 ± 59.3	0.861	113 ± 37.2	122 ± 24.4	0.47
Number of cells	24 (from 4 mice)	24 (from 4 mice)		12 (from 4 mice)	12 (from 4 mice)	
**Number of processes (200–300 μm)**						
Mean ± SD	3.13 ± 0.80	3.25 ± 0.90	0.61	2.92 ± 0.90	3.08 ± 0.52	0.58
Number of cells	24 (from 4 mice)	24 (from 4 mice)		12 (from 4 mice)	12 (from 4 mice)	
**Number of branches (200–300 μm)**						
Mean ± SD	5.63 ± 2.98	5.96 ± 3.59	0.73	7.25 ± 3.19	7.00 ± 2.34	0.83
Number of cells	24 (from 4 mice)	24 (from 4 mice)		12 (from 4 mice)	12 (from 4 mice)	

P10						
Mouse, *P* value	E12 Ctrl	E12 MIA	*P* value	E15 Ctrl	E15 MIA	*P* value
**Soma area (100–200 μm)**						
Mean ± SD (μm^2^)	47.6 ± 9.58	49.3 ± 8.24	0.50	45.1 ± 6.45	46.8 ± 8.16	0.42
Number of cells	26 (from 4 mice)	26 (from 4 mice)		25 (from 4 mice)	25 (from 4 mice)	
**Density (100–200 μm)**						
Mean ± SD (cells/mm^2^)	562 ± 46.4	571 ± 41.4	0.66	601 ± 35.8	581 ± 32.6	0.27
Number of fields	8 (from 4 mice)	8 (from 4 mice)		8 (from 4 mice)	8 (from 4 mice)	
**Total process length (100–200 μm)**						
Mean ± SD (μm)	314 ± 102	276 ± 60.4	0.24	266 ± 14.9	266 ± 40.9	1.00
Number of cells	14 (from 4 mice)	14 (from 4 mice)		12 (from 4 mice)	12 (from 4 mice)	
**Number of processes (100–200 μm)**						
Mean ± SD	4.93 ± 1.00	4.50 ± 0.94	0.25	4.67 ± 0.65	4.58 ± 0.67	0.76
Number of cells	14 (from 4 mice)	14 (from 4 mice)		12 (from 4 mice)	12 (from 4 mice)	
**Number of branches (100–200 μm)**						
Mean ± SD	33.4 ± 15.8	29.3 ± 6.07	0.38	35.6 ± 5.05	37.0 ± 6.08	0.54
Number of cells	14 (from 4 mice)	14 (from 4 mice)		12 (from 4 mice)	12 (from 4 mice)

**Table 3:** Cytokines analysis of P10 microglia.

Liver	E12 Ctrl	E12 MIA	*P*	*N*	E15 Ctrl	E15 MIA	*P*	*N*
CD68	0.19 ± 0.059	0.12 ± 0.026	0.063	4 vs. 4 (4 samples from 4 mice)	0.24 ± 0.027	0.16 ± 0.12	0.29	3 vs. 4 (3 samples from 3 mice, 4 samples from 4 mice)
ICAM-1	3.9 × 10^−3^ ± 1.6 × 10^−3^	3.2 × 10^−3^ ± 0.40 × 10^−3^	0.43	4 vs. 4 (4 samples from 4 mice)	4.9 × 10^−3^ ± 1.3 × 10^−3^	6.9 × 10^−3^ ± 3.7 × 10^−3^	0.43	3 vs. 4 (3 samples from 3 mice, 4 samples from 4 mice)
IL-17a	0.86 × 10^−4^ ± 0.61 × 10^−4^	2.0 × 10^−4^ ± 1.8 × 10^−4^	0.27	4 vs. 4 (4 samples from 4 mice)	0.64 × 10^−4^ ± 1.2 × 10^−4^	0.84 × 10^−4^ ± 0.58 × 10^−4^	0.77	4 vs. 4 (4 samples from 4 mice)
Sall1	0.11 ± 0.058	0.071 ± 0.024	0.27	4 vs. 4 (4 samples from 4 mice)	0.090 ± 0.038	0.14 ± 0.033	0.15	3 vs. 4 (3 samples from 3 mice, 4 samples from 4 mice)
P2Y12R	0.24 ± 0.073	0.13 ± 0.049	0.024	5 vs. 5 (5 samples from 5 mice)	0.18 ± 0.041	0.35 ± 0.16	0.13	3 vs. 4 (3 samples from 3 mice, 4 samples from 4 mice)
TMEM119	7.1 × 10^−2^ ± 3.5 × 10^−2^	7.6 × 10^−2^ ± 2.2 × 10^−2^	0.83	4 vs. 4 (4 samples from 4 mice)	4.9 × 10^−2^ ± 3.0 × 10^−2^	12 × 10^−2^ ± 1.7 × 10^−2^	0.0078	4 vs. 4 (4 samples from 4 mice)

### MIA increases motility of fetal microglial

**Figure 3:**
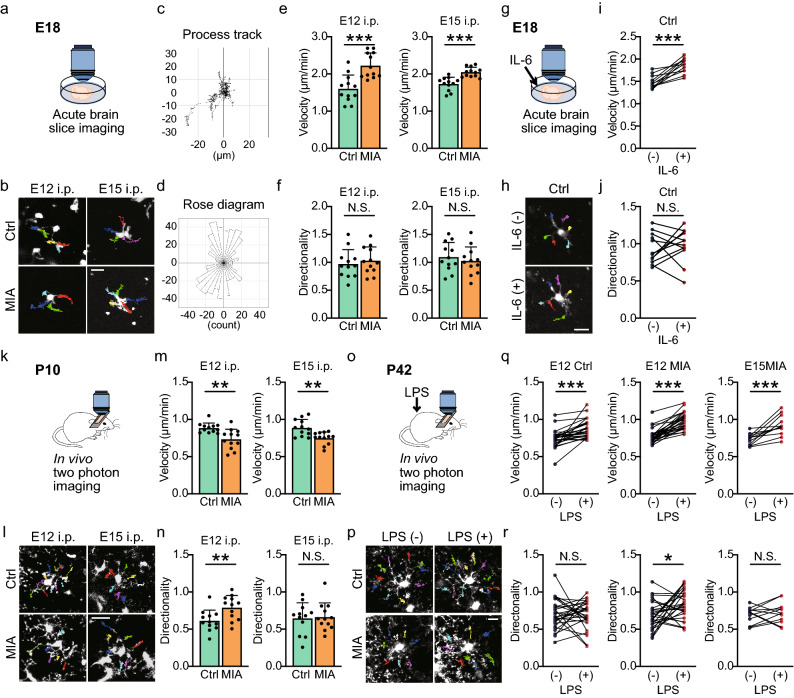
MIA induces sustained changes in patterns of microglial process motility. (**a**) Experimental scheme to illustrate imaging of microglia in the somatosensory cortex in brain slices isolated from E18 offspring of E12 or E15 control (Ctrl; saline -injected) or MIA (Poly(I:C) -injected) mice. (**b**) Representative trajectories of E18 microglial processes from prior control or MIA (E12 and E15) mice. (**c**) A typical microglia process track showing the trajectory of each process tip movement relative to its starting point. (**d**) A tip process track was binned into a Rose diagram using the Migration Tool 2.0 (IBID) to give a directionality profile. Each bin represents the number of process tip tracks moving in that direction. To compare the directionality, the coefficient of variation of each bin (every 10 degrees value) was calculated. (**e**, **f**) Averaged microglial process tip movement velocity (**e**) and directionality (**f**) for microglia in E18 brains isolated from E12 or E15 Ctrl or MIA mice (*n* = 12 cells from 4 mice in each group). (**g**) The inflammatory cytokine, IL-6, was directly added to E18 fetal brain slices from control mice. (**h**) Representative trajectories of microglial processes from E18 slices without and with IL-6 added. (**i**, **j**) Averaged effects of IL-6 on microglial tip process velocity and directionality (*n* = 12 cells from 4 mice in each group). Data from each experiment is shown connected by a line to represent values before and after IL6 application. (**k**) Experimental scheme showing in vivo two-photon imaging of cortical microglia in P10 offspring. (**l**) Representative trajectories of the processes of microglia in a P10 offspring from E12 or E15 saline (Ctrl) or Poly(I:C) (MIA) -injected) mice. Each process from single microglia has a distinct color. (**m**, **n**) Averaged microglial process velocity (**m**) and directionality (**n**) (*n* = 12 cells from 4 mice in each group). (**o**) Schema demonstrating in vivo two-photon imaging of microglia in somatosensory cortex of P42 mice before and after LPS injection. (**p**) Representative trajectories of microglial processes in P42 offspring from E12 saline-injected (Ctrl) or Poly(I:C) -injected (MIA) mice before and after systemic injection of LPS. (**q**, **r**) Grouped effects of LPS on microglial process velocity (**q**) and directionality (**r**) in E12 Ctrl and MIA mice, and E15 MIA mice (E12: *n* = 28 cells from 8 mice; E15: *n* = 12 cells from 4 mice). For all column graphs, columns represent mean ± standard deviation, while dots show data from each process. For (**q**) and (**r**), each experimental data point is shown with values before and after LPS connected by lines. *P < 0.05, **P < 0.01, ***P < 0.001 and N.S.: not significant, unpaired or paired *t*-test.

The function of microglia in vivo depends critically on the motility of their processes, both in surveying brain parenchyma and in the directed migration preceding phagocytosis. Hence, to further characterize how MIA effects microglial phenotypes, we quantified process motility in brain slices acutely isolated from E12 and E15 Poly(I:C) -injected CX_3_CR1-EGFP mice using two-photon microscopy (Fig. 3a–f). We targeted microglia 200–300 μm below the brain slice surface to minimize any effects of acute inflammation resulting from cells injured at the surface of the brain slices. The velocity of the tips of microglial processes were analyzed by tracking their location every minute and quantifying the total distance moved over time, while tip trajectories were quantified using rose diagrams to represent the frequency of process migration angles, which we called “directionality” (see material and methods) and where a value of 1 equates to total movements being symmetrical. The combination of tip velocity and tip directionality reflect the microglial surveillance patterns in brain parenchyma (Fig. 3a–d). Maternal injection of Poly(I:C) at both E12 and E15 increased the velocity of microglial tip movements (Fig. 3e, Table 4), while there were no detectable changes in directionality in either E12 or E15 Poly(I:C) -injected mice (Fig. 3f, Table 4). Finally, we examined how the MIA effects compared to effects of acute application of cytokines to control microglia. Microglial movements in control brain slices with and without addition of exogenous IL-6 were compared (Fig. 3g–j). Acute application of IL-6 had the same effects as was seen with MIA, an increase in microglial process velocity but no change in directionality (Fig. 3i, j, Table 4).

**Table 4:** Process motility analysis of microglia at E18, P10 and P42.

E18						
Mouse, *P* value	E12 Ctrl	E12 MIA	*P* value	E15 Ctrl	E15 MIA	*P* value
**Velocity**						
Mean ± SD (μm/min)	1.60 ± 0.37	2.23 ± 0.34	0.0003	1.73 ± 0.18	2.05 ± 0.13	< 0.0001
Number of cells	12 (from 4 mice)	12 (from 4 mice)		12 (from 4 mice)	12 (from 4 mice)	
**Directionality**						
Mean ± SD	0.97 ± 0.26	1.03 ± 0.24	0.56	1.10 ± 0.26	1.03 ± 0.25	0.51
Number of cells	12 (from 4 mice)	12 (from 4 mice)		12 (from 4 mice)	12 (from 4 mice)	

E18						
IL-6, *P* value	(−)	(+)	*P* value	(−)	(+)	*P* value
	**Velocity**			**Directionality**		
Mean ± SD (μm/min)	1.49 ± 0.14	1.86 ± 0.16	< 0.0001	0.94 ± 0.20	0.98 ± 0.23	0.59
Number of cells	12 (from 4 mice)	12 (from 4 mice)		12 (from 4 mice)	12 (from 4 mice)	

P10						
Mouse, *P* value	E12 Ctrl	E12 MIA	*P* value	E15 Ctrl	E15 MIA	*P* value
**Velocity**						
Mean ± SD (μm/min)	0.89 ± 0.0067	0.73 ± 0.13	0.0020	0.89 ± 0.11	0.75 ± 0.08	0.0022
Number of cells	12 (from 4 mice)	12 (from 4 mice)		12 (from 4 mice)	12 (from 4 mice)	
**Directionality**						
Mean ± SD	0.61 ± 0.14	0.79 ± 0.16	0.0098	0.65 ± 0.21	0.66 ± 0.19	0.82
Number of fields	12 (from 4 mice)	12 (from 4 mice)		12 (from 4 mice)	12 (from 4 mice)	

P42						
LPS, *P* value	Before	After	*P* value	Before	After	*P* value
**E12 Ctrl**						
	**Velocity**			**Directionality**		
Mean ± SD (μm/min)	0.74 ± 0.12	0.87 ± 0.12	< 0.0001	0.70 ± 0.19	0.69 ± 0.18	0.88
Number of cells	28 (from 8 mice)	28 (from 4 mice)		28 (from 8 mice)	28(from 8 mice)	
**E12 MIA**						
	**Velocity**			**Directionality**		
Mean ± SD (μm/min)	0.78 ± 0.10	0.98 ± 0.11	< 0.0001	0.69 ± 0.18	0.83 ± 0.19	0.0123
Number of cells	28 (from 8 mice)	28 (from 8 mice)		28 (from 8 mice)	28 (from 8 mice)	
**E15 MIA**						
	**Velocity**			**Directionality**		
Mean ± SD (μm/min)	0.73 ± 0.077	0.92 ± 0.14	0.0007	0.69 ± 0.11	0.72 ± 0.14	0.48
Number of cells	12 (from 4 mice)	12 (from 4 mice)		12 (from 4 mice)	12 (from 4 mice)	

### Effects of MIA on microglial process motility are observed postnatally

An important question was whether MIA had sustained effects on microglia after birth. To test this hypothesis, we used two-photon in vivo imaging in CX_3_CR1-EGFP mice, at P10 and at P42 (Fig. 3k–r). For technical reasons, we imaged P10 mice immediately after the cranial window surgery, whereas P42 animals were imaged two weeks after the surgery. Again, we imaged microglia from between 100 μm to 200 μm beneath the brain surface, to minimize any effects of acute surface inflammation. In addition, we quantified the microglial morphology just after the cranial surgery (using in vivo imaging) and compared these parameters to that of in the fixed tissue (where there was no cranial surgery). The density of microglia, soma size, total process length, number of processes and number of branches were again not different in E12 control mice, E12 MIA mice, E15 control mice and E15 MIA mice (Supplementary Fig. S2 a–l, Table 5). Nor were there differences between morphology in fixed and live tissues, indicating no detectable influence of the craniotomy operation (Supplementary Fig. S2 a–l, Table 5). However, in contrast to the direction of the change observed in E18 slices, the velocity of microglial tip movements was lower in P10 animals with prior MIA, for both E12 and E15 Poly(I:C) -injections (Fig. 3m, Table 4). In E12 Poly(I:C) -injected mice, the directionality of microglia process movements was increased, suggesting a more repetitive or targeted movement pattern. This directionality change was not seen in E15 Poly(I:C) -injected mice (Fig. 3n, Table 4). In addition, the fluorescent variation in primary process of microglia was larger in E12 and E15 MIA mice compared with each control, suggesting some of the microglial properties change in microglia (Supplementary Fig. S2 m, n).

**Table 5:** Comparison of morphological properties between P10 microglia with and without surgery.

	E12 Ctrl			E12 MIA		
Surgery, *P* value	(−)	(+)	*P* value	(−)	(+)	*P* value
	**Soma area (μm**^**2**^**)**			**Soma area (μm**^**2**^**)**		
Mean ± SD	47.6 ± 9.58	47.9 ± 7.37	0.21	49.3 ± 8.24	47.3 ± 7.37	0.49
Number of cells	26 (from 4 mice)	12 (from 4 mice)		26 (from 4 mice)	12 (from 4 mice)	
	**Density (cells/mm**^**2**^**)**			**Density (cells/mm**^**2**^**)**		
Mean ± SD	562 ± 46.4	573 ± 27.6	0.57	571 ± 41.4	551 ± 52.2	0.39
Number of fields	8 (from 4 mice)	8 (from 4 mice)		8 (from 4 mice)	8 (from 4 mice)	
	**Total process length (μm)**			**Total process length (μm)**		
Mean ± SD	314 ± 102	300 ± 42.2	0.66	276 ± 60.4	298 ± 42.9	0.29
Number of cells	14 (from 4 mice)	12 (from 4 mice)		14 (from 4 mice)	12 (from 4 mice)	
	**Number of processes**			**Number of processes**		
Mean ± SD	4.93 ± 1.00	4.75 ± 0.45	0.57	4.50 ± 0.94	4.58 ± 0.51	0.79
Number of cells	14 (from 4 mice)	12 (from 4 mice)		14 (from 4 mice)	12 (from 4 mice)	
	**Number of branches**			**Number of branches**		
Mean ± SD	33.4 ± 15.8	31.3 ± 4.01	0.67	29.3 ± 6.07	29.0 ± 3.01	0.88
Number of cells	14 (from 4 mice)	12 (from 4 mice)		14 (from 4 mice)	12 (from 4 mice)	

	E15 Ctrl			E15 MIA		
Surgery, *P* value	(−)	**(+)**	*P* value	(−)	**(+)**	*P* value
	**Soma area (μm**^**2**^**)**			**Soma area (μm**^**2**^**)**		
Mean ± SD	45.1 ± 6.45	44.5 ± 5.60	0.76	46.8 ± 8.16	45.4 ± 8.22	0.63
Number of cells	25 (from 4 mice)	12 (from 4 mice)		25 (from 4 mice)	12 (from 4 mice)	
	**Density (cells/mm**^**2**^**)**			**Density (cells/mm**^**2**^**)**		
Mean ± SD	601 ± 35.8	581 ± 36.9	0.30	581 ± 32.6	559 ± 43.9	0.27
Number of fields	8 (from 4 mice)	8 (from 4 mice)		8 (from 4 mice)	8 (from 4 mice)	
	**Total process length (μm)**			**Total process length (μm)**		
Mean ± SD	266 ± 14.9	277 ± 41.6	0.36	266 ± 40.9	275 ± 43.9	0.60
Number of cells	12 (from 4 mice)	12 (from 4 mice)		12 (from 4 mice)	12 (from 4 mice)	
	**Number of processes**			**Number of processes**		
Mean ± SD	4.67 ± 0.65	4.83 ± 0.58	0.51	4.58 ± 0.67	4.33 ± 0.65	0.36
Number of cells	12 (from 4 mice)	12 (from 4 mice)		12 (from 4 mice)	12 (from 4 mice)	
	**Number of branches**			**Number of branches**		
Mean ± SD	35.6 ± 5.05	33.0 ± 3.74	0.17	37.0 ± 6.08	33.8 ± 4.37 12 (from 4 mice)	0.15
Number of cells	12 (from 4 mice)	12 (from 4 mice)		12 (from 4 mice)		

We next examined the effects of both E12 and E15 Poly(I:C) -injection on microglial process motility in late adolescence (P42). No differences in baseline tip velocity or directionality were observed (Supplementary Fig. S3, Table 6). We also examined the response to an acute inflammatory challenge in P42 mice, using lipopolysaccharide (LPS) injections (1 mg/kg, *i.p.*) which is known to transform microglia^33,34^. LPS injection caused a series of expected morphological changes to microglia in E12 saline -injected control mice; cell soma areas were significantly increased, total process length was shortened, and the number of branches was reduced^34–36^. The exact same pattern of changes was seen in E12, and E15 Poly(I:C) -injected mice (Supplementary Fig. S4, Table 7). Acute LPS also caused a similar extent of increase in the microglial tip velocity across all three mice groups (Fig. 3q). However, the effects on directionality differed, in control and E15 Poly(I:C) -injected mice there was no change with LPS, but E12 Poly(I:C) -injected mice showed both higher velocity and directionality after LPS injection (Fig. 3q, r, Table 4), indicating a subtle difference in the characteristics of microglial processes motility following Poly(I:C) -injection at E12.

**Table 6:** Comparison of velocity and directionality among P42 microglia from E12 saline -injected, E12 Poly(I:C) -injected and E15 Poly(I:C) -injected mice before LPS.

P42			
Mouse	E12Ctrl	E12MIA	E15MIA
**Velocity (μm/min) (E12Ctrl vs. E12MIA vs. E15MIA)**			
Mean ± SD	0.74 ± 0.12	0.78 ± 0.095	0.73 ± 0.077
Number of cells	28 (from 8 mice)	28 (from 8 mice)	12 (from 4 mice)
	***P value***		
E12Ctrl vs. E12MIA	0.50		
E12Ctrl vs. E15MIA	0.99		
E12MIA vs. E15MIA	0.51		
**Directionality (E12Ctrl vs. E12MIA vs. E15MIA)**			
Mean ± SD	0.70 ± 0.19	0.69 ± 0.18	0.69 ± 0.11
Number of cells	28 (from 8 mice)	28 (from 8 mice)	12 (from 4 mice)
	***P value***		
E12Ctrl vs. E12MIA	0.98		
E12Ctrl vs. E15MIA	0.99		
E12MIA vs. E15MIA	> 1.00

**Table 7:** Morphological analysis of P42 microglia with and without LPS application.

E12 Ctrl						
LPS, *P* value	Before	After	*P* value	Before	After	*P* value
	**Soma area (μm**^**2**^**)**			**Total process length (μm)**		
Mean ± SD	39.5 ± 5.54	45.8 ± 7.69	0.0003	203 ± 31.0	170 ± 37.2	< 0.0001
Number of cells	12 (from 4 mice)	12 (from 4 mice)		12 (from 4 mice)	12 (from 4 mice)	
	**Number of processes**			**Number of branches**		
Mean ± SD	5.08 ± 0.67	5.00 ± 0.60	0.34	14.1 ± 2.58	11.5 ± 3.37	0.0001
Number of cells	12 (from 4 mice)	12 (from 4 mice)		12 (from 4 mice)	12 (from 4 mice)	

E12 MIA						
LPS, *P* value	Before	After	*P* value	Before	After	*P* value
	**Soma area (μm**^**2**^**)**			**Total process length (μm)**		
Mean ± SD	40.4 ± 10.0	46.9 ± 11.9	0.0005	217 ± 35.7	177 ± 30.1	0.0001
Number of cells	12 (from 4 mice)	12 (from 4 mice)		12 (from 4 mice)	12 (from 4 mice)	
	**Number of processes**			**Number of branches**		
Mean ± SD	4.67 ± 0.49	4.58 ± 0.52	0.34	15.0 ± 2.05	11.9 ± 2.81	< 0.0001
Number of cells	12 (from 4 mice)	12 (from 4 mice)		12 (from 4 mice)	12 (from 4 mice)	

E15 MIA						
LPS, *P* value	Before	After	*P* value	Before	After	*P* value
	**Soma area (μm**^**2**^**)**			**Total process length (μm)**		
Mean ± SD	39.8 ± 3.80	47.0 ± 5.25	0.0011	211 ± 40.0	168 ± 29.2	< 0.0001
Number of cells	12 (from 4 mice)	12 (from 4 mice)		12 (from 4 mice)	12 (from 4 mice)	
	**Number of processes**			**Number of branches**		
Mean ± SD	4.75 ± 0.62	4.67 ± 0.49	0.59	15.0 ± 2.13	11.5 ± 1.83	0.0009
Number of cells	12 (from 4 mice)	12 (from 4 mice)		12 (from 4 mice)	12 (from 4 mice)	

### MIA induces sustained behavioral differences in late adolescent offspring

**Figure 4:**
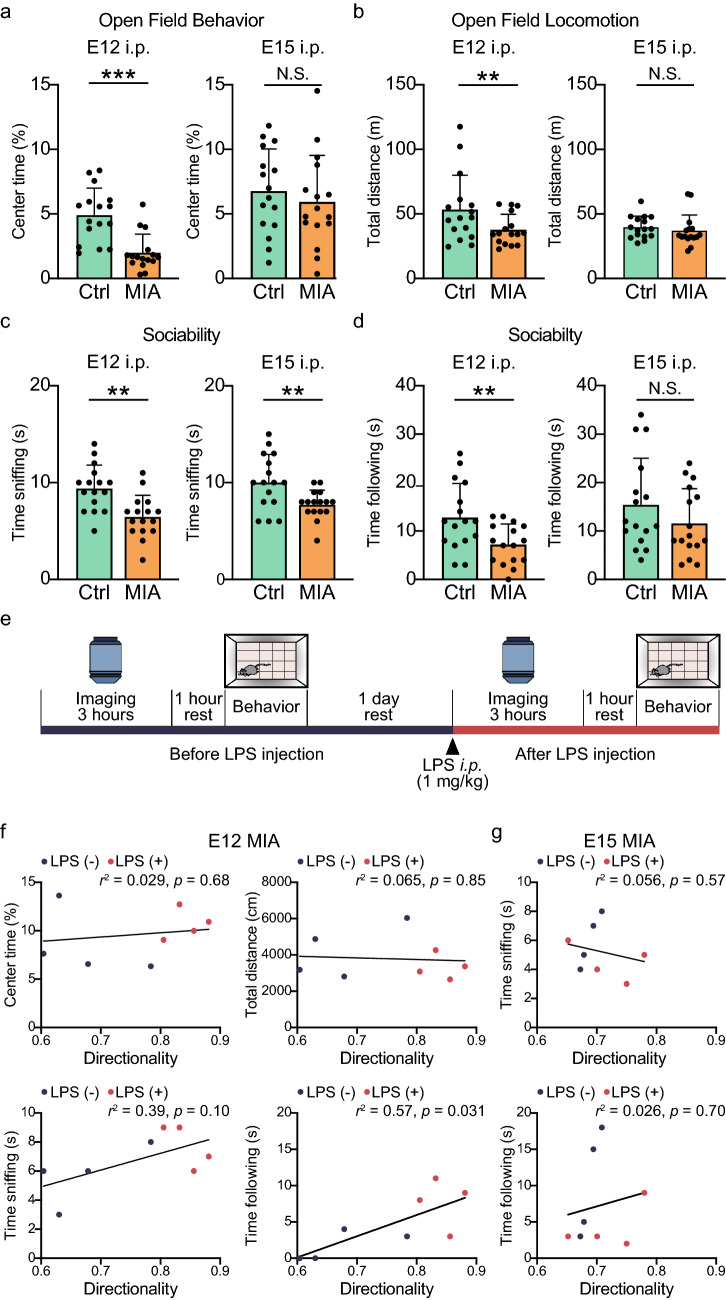
MIA induces sustained changes in behavior that can show weak correlations with microglial tip process directionality. (**a**, **b**) Averaged effects of MIA induced at E12 or E15 on (**a**) the proportion of time spent in the center of the open field or (**b**) on the total distance traveled in the open-field test (*n* = 16 mice in each group). (**c**, **d**) Averaged effects of MIA induced at E12 or E15 on (**c**) the time spent sniffing, or (**d**) following, a novel mouse in the social interaction test (*n* = 16 mice in each group). Columns represent the mean ± standard deviation, while dots show data from each mouse. *P < 0.05, **P < 0.01, ***P < 0.001 and N.S.: not significant, unpaired *t*-test. (**e**) Experimental scheme showing two-photon imaging and behavioral assay before and after LPS injection. (**f**, **g**) Plot of the relationship between behavioral phenotypes and microglial directionality in individual P42 offspring from E12 (**f**) and E15 (**g**) MIA mice, before (black dots) and after (red dots) LPS injection. The regression line plots the linear correlation between the two variables; the time spent following was significantly correlated with directionality (*P* = 0.031, *r* = 0.75, *n* = 8 mice, Pearson’s correlation test). For all plots, *n* = 4 mice before LPS and 4 mice after LPS.

The sustained effects of MIA on microglial motility were subtle, and hence we wished to validate that E12 and E15 Poly(I:C) -injected mice could induce sustained behavioral deficits. In the open field behavior assay, we found reductions in the percent time spent in the center of the field in mice with E12 Poly(I:C) -injection, while no differences were seen for mice with the E15 Poly(I:C) -injections (Fig. 4a, Table 8). The total distance traveled in the open field was also less in E12 Poly(I:C) -injected mice, but not in E15 Poly(I:C) -injected mice (Fig. 4b, Table 8). In a social interaction assay, a naive same-sex mouse was placed in the same cage as the unfamiliar mouse for 10 min. Mice sniff and follow the unfamiliar mouse as social behavior^37^, and we quantified these two aspects of social interaction of control and MIA (E12 and E15) mice. The sniffing time was decreased in both E12 and E15 Poly(I:C) -injected mice compared with that of the control mice (Fig. 4c, Table 8). The time spent following the unfamiliar mouse was significantly decreased in the E12 Poly(I:C) -injected mice, but not in the E15 Poly(I:C) -injected mice (Fig. 4d, Table 8). Finally, we looked for correlation between the behavioral changes in each mouse, and the measurements of microglial directionality in E12 and E15 Poly(I:C) -injected mice both before and after LPS injection (Fig. 4e). In E12 Poly(I:C) -injected mice, the time spent following the unfamiliar mouse directly correlated with changes in directionality (Fig. 4f, bottom right panel, Table 8). There was no relationship between directionality and the percent of time in the center of the open field or with the distance traveled, or with the time spent sniffing (Fig. 4f, Table 8). In E15 Poly(I:C) -injected mice, there was no correlation between social behavior and microglial directionality (Fig. 4g, Table 8). Our result confirms that our MIA models induce sustained behavioral effects, particularly in E12 Poly(I:C) -injected mice, and that the extent of some of these behavioral assays correlates with the extent of changes in microglial directionality.

**Table 8:** Behavioral analysis in late adolescent offspring.

Mouse, *P* value	E12 Ctrl	E12 MIA	*P* value	E15 Ctrl	E15 MIA	*P* value
**Center time**						
Mean ± SD (%)	4.9 ± 2.1	2.0 ± 1.4	< 0.001	6.8 ± 3.3	5.9 ± 3.6	0.50
Number of mice	16	16		16	16	
**Total distance**						
Mean ± SD (m)	53.4 ± 26.5	37.7 ± 11.9	0.0037	39.6 ± 3.3	36.9 ± 3.6	0.48
Number of mice	16	16		16	16	
**Time sniffing**						
Mean ± SD (s)	9.4 ± 2.4	6.5 ± 2.2	0.001	10.1 ± 2.8	7.8 ± 1.5	0.007
Number of mice	16	16		16	16	
**Time following**						
Mean ± SD (s)	12.9 ± 6.9	7.3 ± 4.1	0.010	15.5 ± 9.6	11.7 ± 7.1	0.20
Number of mice	16	16		16	16	

E12 MIA						
**Correlation**	**Directionality vs. center time**	**Pearson r**	***P value***			
Number of mice	8 (before LPS: 4 mice, after LPS: 4mice)	0.17	0.68			
**Correlation**	**Directionality vs. total distance**	**Pearson r**	***P value***			
Number of mice	8 (before LPS: 4 mice, after LPS: 4mice)	-0.080	0.85			
**Correlation**	**Directionality vs. time sniffing**	**Pearson r**	***P value***			
Number of mice	8 (before LPS: 4 mice, after LPS: 4mice)	0.62	0.10			
**Correlation**	**Directionality vs. time following**	**Pearson r**	***P value***			
Number of mice	8 (before LPS: 4 mice, after LPS: 4mice)	0.75	0.031			

E15MIA						
**Correlation**	**Directionality vs. center time**	**Pearson r**	***P value***			
Number of mice	8 (before LPS: 4 mice, after LPS: 4mice)	− 0.24	0.57			
**Correlation**	**Directionality vs. total distance**	**Pearson r**	***P value***			
Number of mice	8 (before LPS: 4 mice, after LPS: 4mice)	0.16	0.70			

## Discussion

In this study, we aimed to identify whether altered microglial properties may be associated with MIA. We induced MIA at two different times in gestation, E12 corresponding to the second trimester and E15, corresponding to third trimester. Maternal inflammation at different gestational periods results in different predispositions to human disease^38^ and to different cognitive deficits in rodents. Offspring from E12 Poly(I:C) -injected rodents, for example, show deficits in latent inhibition and pre-pulse inhibition (PPI)^39^, while E15 Poly(I:C) -injected offspring have normal PPI^40^. The timing of the MIA can also affect microglial responses. E12 Poly(I:C) -injection increased postnatal microglial cytokine and chemokine expression in the offspring, but did not change microglial morphology and density^39,41,42^. In contrast, E15 Poly(I:C) -injection did cause morphological changes in microglia^40^. We first evaluated the cytokine response after our MIA models at mid and late gestation, focusing on expression levels of a series of six pro-inflammatory cytokines (IL-6, IL-1β, IL-17a, TNFα) or cell-surface molecules (CD68 and ICAM-1). These cytokines were selected to include those (IL-6, IL-1β, TNFα) that can be increased in the serum of patients with schizophrenia or autism spectrum disorders^43–46^. Both MIA models caused an inflammatory response in both maternal organs and in the fetal brain. IL-6 is a key mediator of MIA sequelae^12^ and was increased in fetal microglia. Other inflammatory molecules IL-1β and TNFα showed differential responses—IL-1β in fetal microglia increased when MIA occurred at later gestation, while TNFα in fetal microglia decreased only with the earlier MIA. This could be due to a transient change in expression patterns, with IL-1β increasing 3 days after MIA and TNFα decreasing at 6 days after MIA, or it may reflect different responses of E12 and E15 microglia to MIA. A different response of E12 and E15 microglia is consistent with differentiation into a more mature phenotype beginning between these two ages^47,48^. Regardless, our results show that fetal microglia respond, at a biochemical level at least, to maternal inflammation at both mid (E12) and later (E15) gestation.

We next examined morphological changes. Immature microglia display an ameboid morphology during gestation that gradually becomes more ramified over postnatal development^49^. We also observed increases in total process length, and in the number of processes and branches between E18 and P10 (Table 9). However, at both ages, there was no difference in morphology between control and MIA mice, both for E12 and E15 Poly(I:C) -injections. This contrasts to the biochemical phenotypes, and our inability to detect any differences after MIA may partly relate to the already ameboid morphology of microglia at embryonic and early postnatal ages^50–53^. Reported MIA induced changes in microglial density and morphology are very variable, although quite frequently no change has been observed across different studies^42^. We here confirm the qualitative observations that late gestation Poly (I:C) induces no change in microglia morphology, and extend that to mid gestation (E12) MIA^54^. It appears that morphology is a poor measure of changes in microglia phenotype after MIA. In contrast, we show here that microglial process motility is a more sensitive measure of prior MIA. Some aspect of tip motility, either basal tip velocity or directionality, or the response to LPS, was altered by prior MIA. In developing and adult brains, microglial processes are constantly motile, sampling brain parenchyma and contacting soma, synapses, glia and extracellular spaces^55–57^. This is an important part of their physiological function-immune surveillance to enable rapid responses to brain damage^58^ and also sculpting circuits during development via synapse formation or elimination in an activity-dependent fashion^13,14,59–62^. Microglial surveillance may also be important for neural circuit plasticity in the healthy adult brain^63^. Thus, MIA induced changes in microglial tip motility, it may not only effect how synapses are formed and pruned during development but may have ongoing effects on immune-surveillance and neural circuit homeostasis. Just how the altered motility parameters may specifically affect microglial functions is speculative without further follow-up studies. The increase in tip velocity in E18 microglia is at a developmental stage when microglia are proliferating and migrating to the growing cortices and this motility may in part be mediated via trophic signals^48^. An increase in tip velocity may be predicted to enhance migration, although this doesn’t seem to manifest as clear increases in cortical densities in latter postnatal brains^42^. At P10, we observed (for the E12 Poly(I:C) -injected mice), decreases in tip velocity and increases in directionality. At P10 microglia are actively interacting with neurons and potentially both forming and pruning synapses, as well as assisting with apoptosis. The decrease tip velocity may suggest brief pauses during brain surveillance, while the increase directionality may suggest more frequent back and forth movements to single targets. Together this may indicate increased microglial-neuron interactions, something that could ultimately impact postnatal synapse numbers and which could be more directly examined with future in vivo imaging. It was interesting that the directionality changes at P10 were only seen after E12 MIA, and the subsequent behavioral phenotypes were also more severe after E12 MIA. At P42, akin to adolescence, changes in basal microglial motility parameters after MIA were not observed, but different LPS-induced motility patterns occurred after prior MIA. LPS increased tip velocity in all mice cohorts, as seen previously^64^, but mice with E12 MIA also showed an increase in directionality after LPS. We don’t believe this has been noted before, but may again reflect more repetitive interactions with synaptic elements. Although speculative, this may be associated with improved cognitive symptoms sometimes observed in patients with autism during fever and systemic inflammation^65,66^. In the offspring of MIA mice, LPS injection can also reduce the extent of social behavioral deficits, and this may be due to activation of Toll-like receptor subtypes (TLR2, TLR4) on microglia and release of cytokines (IL-17) that can increase social behaviors^67^. We also observed impaired social behaviors in offspring of MIA mice, and particularly for E12 Poly(I:C) mice. Furthermore, in these E12 Poly(I:C) mice there did seem to be some weak relationship between directionality and time spent following the unfamiliar mouse (Fig. 4f). Mice with greater directionality after LPS spent longer following the unfamiliar mouse, suggesting an increased microglial tip process directionality may be associated with improved social behaviors. While this association is speculative, it does suggest it worthwhile to further investigate the physiological significance of directionality and its possible link to how inflammation can increase social behaviors in ASD model mice.

**Table 9:** Comparison of morphological properties between E18 and P10 microglia from control (E12 and E15 saline -injected) and MIA (E12 and E15 Poly(I:C) -injected) mice.

Soma area (μm^2^) (E18 vs. P10)						
Mouse, *P* value	E18	P10	*P* value	E18	P10	*P* value
	**E12 Ctrl**			**E12 MIA**		
Mean ± SD	63.4 ± 15.5	47.6 ± 9.58	< 0.0001	65.7 ± 21.1	49.3 ± 8.24	0.0004
Number of cells	37 (from 4 mice)	26 (from 4 mice)		37 (from 4 mice)	26 (from 4 mice)	
	**E15 Ctrl**			**E15 MIA**		
Mean ± SD	56.6 ± 13.0	45.1 ± 6.45	0.0003	54.8 ± 12.8	46.8 ± 8.16	0.0132
Number of cells	23 (from 4 mice)	25 (from 4 mice)		23 (from 4 mice)	25 (from 4 mice)	

Total process length (μm) (E18 vs. P10)						
Mouse, *P* value	E18	P10	*P* value	E18	P10	*P* value
	**E12 Ctrl**			**E12 MIA**		
Mean ± SD	129 ± 48.9	314 ± 102	< 0.0001	132 ± 59.2	276 ± 60.4	< 0.0001
Number of cells	24 (from 4 mice)	14 (from 4 mice)		24 (from 4 mice)	14 (from 4 mice)	
	**E15 Ctrl**			**E15 MIA**		
Mean ± SD	113 ± 37.2	266 ± 14.9	< 0.0001	122 ± 24.4	266 ± 40.9	< 0.0001
Number of cells	12 (from 4 mice)	12 (from 4 mice)		12 (from 4 mice)	12 (from 4 mice)	

Number of processes (E18 vs. P10)						
Mouse, *P* value	E18	P10	*P* value	E18	P10	*P* value
	**E12 Ctrl**			**E12 MIA**		
Mean ± SD	3.13 ± 0.80	4.93 ± 1.00	< 0.0001	3.25 ± 0.90	4.50 ± 0.94	0.0002
Number of cells	24 (from 4 mice)	14 (from 4 mice)		24 (from 4 mice)	14 (from 4 mice)	
	**E15 Ctrl**			**E15 MIA**		
Mean ± SD	2.92 ± 0.90	4.67 ± 0.65	< 0.0001	3.08 ± 0.51	4.58 ± 0.67	< 0.0001
Number of cells	12 (from 4 mice)	12 (from 4 mice)		12 (from 4 mice)	12 (from 4 mice)	
	**E12 Ctrl**			**E12 MIA**		
Mean ± SD	5.63 ± 2.98	33.4 ± 15.8	< 0.0001	5.96 ± 3.59	29.3 ± 6.07	< 0.0001
Number of cells	24 (from 4 mice)	14 (from 4 mice)		24 (from 4 mice)	14 (from 4 mice)	
	**E15 Ctrl**			**E15 MIA**		
Mean ± SD	7.25 ± 3.19	35.6 ± 5.05	< 0.0001	7.00 ± 2.34	37.0 ± 6.08	< 0.0001
Number of cells	12 (from 4 mice)	12 (from 4 mice)		12 (from 4 mice)	12 (from 4 mice)	

In conclusion, we report that changes in basal or induced microglial tip motility patterns can be a sensitive measure of neuro-immune alterations in the offspring of mothers with infection, and show here that such motility changes can be observed as early as E18 and are sustained through to adolescence (P42). This strengthens the ideas that changes in microglial functions may contribute to the link between maternal infection and subsequent cognitive defects, and susceptibility to schizophrenia, or developmental disorders in the offspring.

## Methods

### Animals

All experiment protocols were approved by the Animal Care and Use Committees of Kobe University Graduate School of Medicine and were conducted according to the guidelines of the National Institutes of Health Guide for the Care and Use of Laboratory Animals. The animals in this study were given free access to food and water and housed in a 12 h light/dark cycle. In order to visualize microglial morphology and motility, we used CX_3_CR1-EGFP transgenic mice expressing enhanced green fluorescent protein (EGFP) under the control of the CX_3_CR1 promoter, specific for microglia and monocytes^68^. We procured heterozygous CX_3_CR1-EGFP fetuses after male CX_3_CR1-EGFP mice (homozygous) were crossed with female C57BL/6J mice^46^.

### Maternal immune activation

Pregnant mice were injected intraperitoneally with 10 mg/kg polyinosinic:polycytidylic acid [Poly(I:C); Sigma-Aldrich, St. Louis, MO)] to induce MIA^69^. Control mice were injected with an equal volume of saline.

### Isolation of fetal microglia, P10 microglia and maternal tissues

Mice on Day 18 of gestation (GD18) and male mice 10 days postpartum (P10) were deeply anesthetized with ketamine and xylazine, and transcardially perfused with PBS. Fetal brains from both males and females, maternal tissues (liver, placenta and brain) and P10 mice brains were extracted and immediately placed in cold PBS. Fetal and P10 mice cortices were minced and dissociated with the Neural Tissue Dissociation Kit (T) (130-093-231, Miltenyi Biotec, Bergisch Gladbach, Germany). Debris was removed by passing samples through a cell strainer (40 μm) with 5% BSA-PBS. Using a magnetic-activated cell sorting (MACS) system, the CD11b positive cells were magnetically labeled with CD11b magnetic beads (for microglia, 130-093-634, Milteny Biotec) and retained in the MS Column (130-042-201, Miltenyi Biotec). The CD11b positive cells were eluted by removing the MS Colum from the magnetic field^70^.

### Real time PCR

To quantify mRNA expression of pro-inflammatory cytokines in microglia and maternal tissues (liver, placenta and brain) and expression of other microglia-specific genes related to differentiation, total RNA was extracted from MACS-isolated microglia and GD18 maternal tissues using RNeasy Plus Mini kit (74134; Qiagen, Hilden, Germany). First-strand complementary DNA (cDNA) was synthesized from total RNA using Transcriptor First Strand cDNA Synthesis Kit (04896866001, Roche Diagnostics, Mannheim, Germany). PCR was performed on LightCycler 96 System (Roche Diagnostics) using the FastStart Essential DNA Green Master (06402712001, Roche Diagnostics). Amplification results were analyzed with the LightCycler software and then normalized based on the GAPDH mRNA levels in each sample.

The following pairs of primers were used:

*IL-6* were forward: 5′-CCACTTCACAAGTCGGAGGCTTA-3′ and reverse: 5′-CCAGTTTGGTAGCATCCATCATTTC-3′; *IL-1β* were forward: 5′-TCCAGGATGAGGACATGAGCAC-3′ and reverse: 5′-GAACGTCACACACCAGCAGGTTA-3′; *TNFα* were forward: 5′-ACTCCAGGCGGTGCCTATGT-3′ and reverse: 5′-GTGAGGGTCTGGGCCATAGAA-3′; *IL-17a* were forward: 5′-TCCAGAAGGCCCTCAGACTA-3′ and reverse: 5′-CTCGACCCTGAAAGTGAAGG-3′; *CD68* were forward: 5′-TGATCTTGCTAGGACCGCTTA-3′ and reverse: 5′-TAACGGCCTTTTTGTGAGGA-3′; *ICAM-1* were forward: 5′-CCTGTTTCCTGCCTCTGAAG-3′ and reverse: 5′-GTCTGCTGAGACCCCTCTTG-3′; *SALL1* were forward: 5′-GACATCCCCAGTTCTGCTCC-3′ and reverse: 5′-ACCTCGCCGCTAGATCCTTC-3′; *P2Y12R* were forward: 5′-CAGGTTCTCTTCCCATTGCT-3′ and reverse: 5′- CAGCAATGATGATGAAAACC-3′; *TMEM119* were forward: 5′-GTGTCTAACAGGCCCCAGAA-3′ and reverse: 5′-AGCCACGTGGTATCAAGGAG-3′; *GAPDH* were forward: 5′-AATGCATCCTGCACCACCAAC-3′ and reverse: 5′-TGGATGCAGGGATGATGTTCTG3′. For the analysis of mRNA expression in E18 microglia, each pool consisted of 2–3 embryo brains.

### Cranial window surgery

We performed surgery for in vivo imaging on P10^71^ and on 4-week-old^72^ male mice. In P10 mice, the skull was exposed and cleaned under isoflurane (1%) anesthesia, and a custom-made head plate was firmly attached to the skull with dental cement (G-CEM ONE; GC, Tokyo, Japan). The head plate allowed us to securely attach the mouse to a stainless frame for both cranial window surgery and the subsequent two-photon imaging. After securing the head plate we performed a circular craniotomy (1.6 mm diameter) over the left somatosensory cortex (S1, centered at 1.0 mm posterior from bregma and 2.5 mm lateral from the midline). A 2 mm glass coverslip was placed over the exposed brain to form a cranial window. The edges of the cranial window were sealed with a combination of adhesive glue (Aron Alpha, Konishi, Osaka Japan) and dental adhesive resin cement (Super Bond; Sun Medical, Shiga, Japan). Imaging began immediately after surgery. For 4-week-old mice, following anesthesia with ketamine (74 mg/kg, *i.p.*) and xylazine (10 mg/kg, *i.p.*), the skull was exposed and cleaned, and a different and larger (custom-made) head plate was similarly attached. One day later, we performed the circular craniotomy (2 mm diameter) over the left primary sensory cortex (S1, centered at 1.5 mm posterior from bregma and 2.5 mm lateral from the midline) under isoflurane (1%) anesthesia. After the craniotomy, 2% (w/v) agarose L dissolved (Nippon Gene, Tokyo, Japan) in saline was applied, and a glass window comprising two coverslips (2 & 4.5 mm each; Matsunami Glass, Osaka, Japan) were placed over the brain surface with ultraviolet curable adhesive (NOR-61, Norland, New Jersey, USA). The edges of the cranial window were sealed with a combination of dental cement and dental adhesive resin cement (Super Bond; Sun Medical, Shiga, Japan). These 4-week mice were returned to their home cages and imaging experiments were performed two weeks later, at P42.

### In vivo two-photon imaging

For both 10-day- and 6-week-old (P42) mice, two-photon imaging was acquired from the left S1 using a laser scanning system (LSM 7 MP system; Carl Zeiss, Oberkochen, Germany) with two types of water-immersion objective lenses (10 ×, numerical aperture (N.A.) 0.5; 20 ×, N.A. 1.0; Carl Zeiss, Germany) and a Ti:sapphire laser (Mai Tai HP; Spectra-Physics, Santa Clara, CA) operating at 950-nm wavelength. Fluorescence was collected using GaAsP photomultiplier tubes (Hamamatsu Photonics, Shizuoka, Japan)^73^.

### Acute slice imaging

To obtain E18 cortical slices, male and female fetal brains were quickly isolated and sliced coronally (500 μm) with a vibratome in cold, oxygenated cutting solution (4 °C, bubbled with 95% O_2_–5% CO_2_). Immediately after sectioning, the slices were warmed in oxygenated recovery solution (32 °C, bubbled with 95% O_2_- 5% CO_2_) for 30 min. Cutting solution contained (in mM) 126 C_5_H_14_CINO, 3.5 KCl, 26 NaHCO_3_, 1.2 NaH_2_PO_4_, 0.5 CaCl_2_, 7.0 MgSO_4_, 10 d-glucose. Recovery solution contained (in mM) 93 NMDG, 2.5 KCl, 30 NaHCO_3_, 1.2 NaH_2_PO_4_, 20 HEPES, 0.5 CaCl_2_, 10.0 MgSO_4_, 25 D-glucose, 5 sodium ascorbate, 3 sodium pyruvate, 12 N-Acetyl-l-cysteine, pH 7.4 (adjusting for HCl). Two-photon images were then acquired from the E18 cortical slice immersed in artificial cerebrospinal fluid (ACSF) (37 °C, bubbled with 95% O_2_–5% CO_2_). ACSF contained (in mM) 126 NaCl, 3.5 KCl, 26 NaHCO_3_, 1.2 NaH_2_PO_4_, 2.0 CaCl_2_, 1.3 MgSO_4_, 10 D-glucose, pH 7.45. Acute slice imaging was acquired using two types of two-photon and one photon laser scanning systems; LSM 7 MP system (Carl Zeiss, Oberkochen, Germany) with water-immersion objective lenses (20 ×, N.A. 1.0; Carl Zeiss, Germany) and a Ti:sapphire laser (Mai Tai HP; Spectra-Physics, Santa Clara, CA) operating at 950-nm wavelength, fluorescence was collected using GaAsP photomultiplier tubes (Hamamatsu Photonics, Shizuoka, Japan), and NIS-Elements (Nikon Instech Co., Ltd, Tokyo, Japan) with a water-immersion objective lens (16 ×, NA 0.80; Nikon Instech Co., Ltd) and a Chameleon Discovery (Coherent, California, United States) setting at 950-nm wavelength, fluorescence was collected using C2 Si (Nikon Instech Co., Ltd); FV10-ASW(Olympus Co., Ltd, Tokyo, Japan) with a water-immersion objective lens (40 ×, NA 0.80; Olympus Co., Ltd.) and an Argon laser (Showa Optronics Co., Ltd.) set at 488-nm wavelength and fluorescence was collected using FLUOVIEW FV1000 (Olympus Co., Ltd, Tokyo, Japan). For the experiment to assess effects of IL-6 (recombinant mouse IL-6 protein, Lot: NUQ3019031, R&D Systems Inc, Minnesota, United States) on microglial motility, acute E18 brain slices were imaged using two types of two-photon systems and one photon laser scanning system 1–2 h before and during IL-6 application, respectively.

### LPS challenge in MIA offspring

Lipopolysaccharide (LPS; Funakoshi, Tokyo, Japan) was administered to induce systemic inflammation in offspring of MIA mice. Single doses of LPS (1.0 mg/kg, *i.p.*) were injected one day after control two-photon imaging in 6-week-old (P42) mice. Two-photon imaging was then started 3 h after LPS injection to assess microglial responses to systemic inflammation. Behavioral assays (see below) were examined in the same mice, just after the control and post-LPS imaging sessions (corresponding to 4–5 h after LPS).

### Confocal imaging

P10 male mice or GD18 mice were deeply anesthetized with ketamine and xylazine, and transcardially perfused with 4% paraformaldehyde solution in PBS. Fixed P10 male mice brains and E18 male and female fetal brains were extracted from the skull and post-fixed overnight in the same solution followed by 30% sucrose. The brains were cut into 50-μm sections with a microtome (Leica Microsystems, Wetzlar, Germany). Fixed tissue imaging was performed using a Zeiss LSM510 Meta confocal microscope (Carl Zeiss, Oberkochen, Germany) with a 20 × objective (NA 1.0; Carl Zeiss) or a 63 × oil-immersion objective (NA 1.4).

### Image analysis

All images were analyzed using MATLAB software (MathWorks, Natick, MA) and ImageJ (National Institutes of Health, Bethesda, MD). Movies were corrected for focal plane displacements using both TurboReg^46^. The E18 microglia in the superficial and deep layers of the somatosensory cortex (0–50 μm and 200–250 μm from the pia) were analyzed in Z-projected images (confocal image; 512 × 512 pixels, 0.397 μm/pixel, 1 μm Z-step [rostrocaudal direction], 21 slices, maximum intensity projection; two-photon image; 512 × 512 pixels, 0.415 μm/pixel, 1 μm Z-step [rostrocaudal direction], 51 slices). The P10 microglia in the somatosensory cortex (100–200 μm from the pia) were analyzed in Z-projected images (confocal image; 512 × 512 pixels, 0.198 μm/pixel, 1 μm Z-step [rostrocaudal direction], 21 slices, maximum intensity projection; two-photon image; 512 × 512 pixels, 0.415 μm/pixel, 1 μm Z-step [dorsoventral direction], 71 slices). The P42 microglia in the somatosensory cortex (100–200 μm from the pia) were analyzed in Z-projected images (two-photon image; 512 × 512 pixels, 0.415 μm/pixel, 1 μm Z-step [dorsoventral direction], 71 slices). To quantify E18 and P10 microglial morphology and P42 microglial morphology before and after LPS administration, the reconstructed Z-stacked confocal images and the reconstructed 20 μm Z-stacked two-photon images were analyzed by using the ImageJ plug-in Simple Neurite Tracer and the segmented line tool in ImageJ respectively. In addition, to assess microglial motility based on the reconstructed Z-stacked two-photon movies (time interval 1 min), we used the ImageJ plug-in Manual Tracking. Using chemotaxis and Migration Tool 2.0 (IBIDI), we measured the velocity and directionality of microglial processes by tracking the process tip every 1minute^74,75^. The rose diagram is a circular histogram which displays the direction or angle that the microglial tip moves, relative to its starting point, and the frequency of each direction. Each direction angle was binned, with 9 bins per quadrant (10° each), and the area of each bin is the number of tip movements in that direction. To compare the directionality statistically, we calculated the coefficient of variation of each bin (every 10 degrees value). An averaged value of 1 for the total microglial tip movements represent symmetrical movements.

### Behavioral tests

Animals were allowed to acclimatize to the behavioral testing room for at least 1 h before testing. 6-week-old juvenile male mice were placed in a 50 × 50 cm white plexiglas box open field and movements were recorded by a video and subsequently analyzed using Duo Mouse software (National Institute of Genetics, Shizuoka, Japan) and MATLAB (MathWorks, Natick, MA) software packages. Mice were tracked by camera (C270N HD WEBCAM, Logicool, Tokyo, Japan) and the total movement over 10 min in the open field quantified. The center of the open field was defined as a central 12.5 × 12.5 square (156.25 cm^2^, 6.25% of the total area) and the total time when mice were tracked as being inside this region quantified. For the socialization assays, a novel 6-week-old unfamiliar male was introduced into the test subject’s arena into the diagonally opposite region for 10 min and the resulting socialization parameters were scored subjectively as one mouse walking closely behind the other, keeping pace, while nose-to-nose sniffing was defined as one mouse closely approaching the other and sniffing its nose. Non-social parameters were scored as arena exploration (walking around the arena, sniffing the walls or floor).

## Supplementary information

Supplementary Figure S1.

Supplementary Figure S2.

Supplementary Figure S3.

Supplementary Figure S4.

## Data Availability

All data were analyzed using GraphPad Prism 8 statistical software (GraphPad Software Inc., La Jolla, CA). All data are presented as means ± SD. Unpaired *t*-test, paired *t*-test, one-way ANOVA and Pearson’s correlation test were used to test for statistical significance.
