# Supplementary material for: Maternal immune activation induces sustained changes in fetal microglia motility
Source: Sci Rep. 2020 Dec 7;10:21378. doi: 10.1038/s41598-020-78294-2 (PMC7721716; doi:10.1038/s41598-020-78294-2)
Supplement: Supplementary file 1 — Supplementary Figure S1. [file 41598_2020_78294_MOESM1_ESM.pdf]

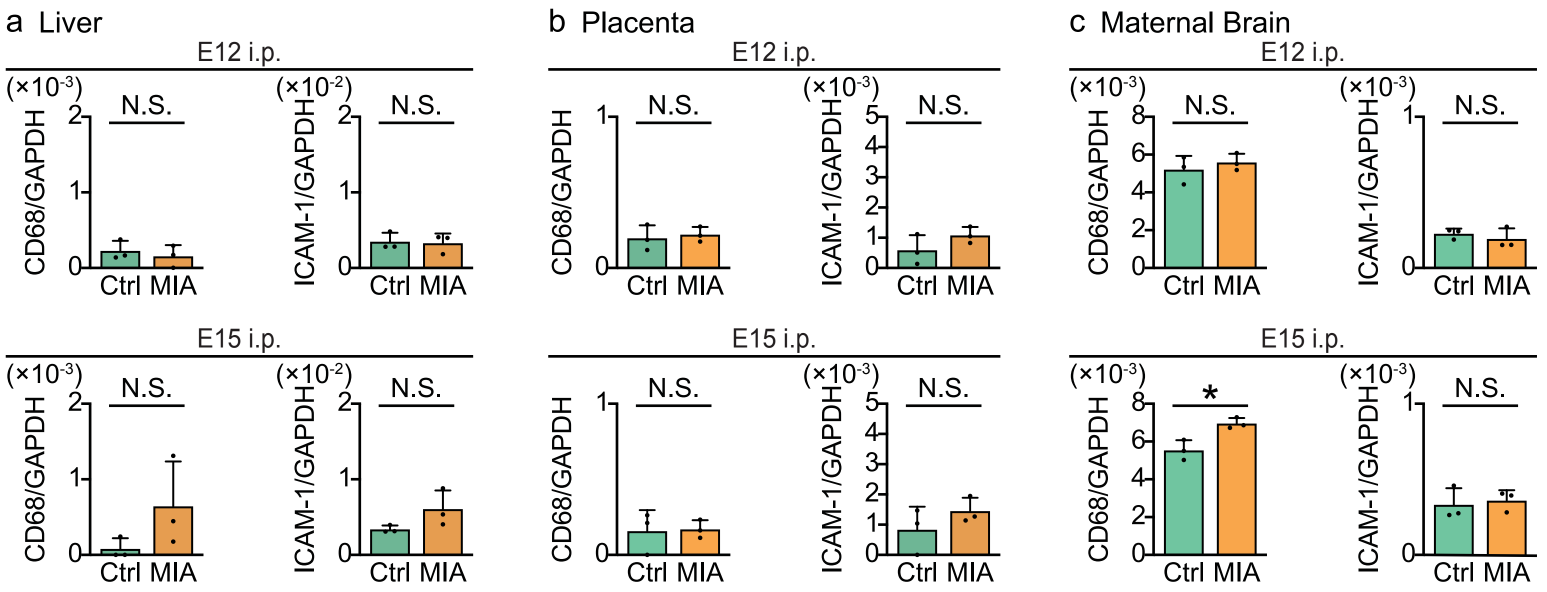

Relative CD68 and ICAM-1 mRNA expression in maternal liver **(a)**, placenta **(b)** and brain **(c)** (n = 3 mice in each group). There were no differences between control vs Poly(I:C) E12 or E15 -injected mice, with the exception of CD68 relative expression in E15 injected mice brains. Columns represent the mean ± standard deviation, while dots show data from each sample. *$P < 0.05$, N.S.: not significant, unpaired *t*-test.

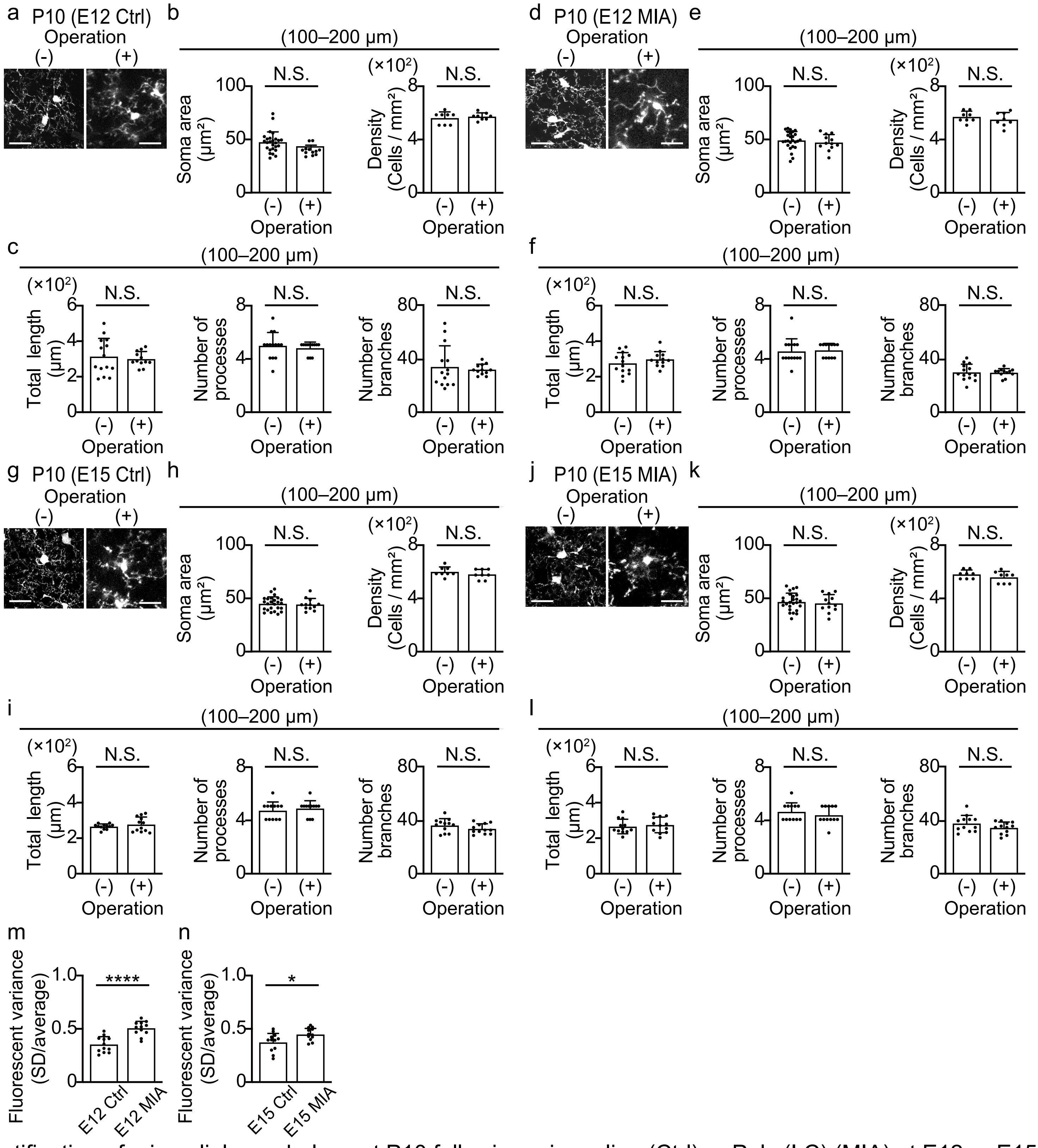

Quantification of microglial morphology at P10 following prior saline (Ctrl) or Poly (I:C) (MIA) at E12 or E15 as indicated. **(a-l)** Images for analysis were obtained both *in vivo*, using two-photon imaging after cranial window surgery (operation (+)) and from *in vitro* fixed tissue using immunohistochemistry (operation (-)). Neither the craniotomy operation, nor the MIA had any effect on morphology in these P10 brains. **(m, n)** Averaged data for variance of primary process intensity at P10 microglia. Panels **(a, d, g, j)** show typical images with higher-magnification insets (Scale bars, 20 μm). Graphs in panels **(b, c, e, f, h, i, k, l, m, n)** show mean and SD of different morphological parameters, dots show data from single samples (a single microglia or a single field for the analysis of density). **P* < 0.05, ****P* < 0.0001, N.S.: not significantly different, unpaired *t*-test.

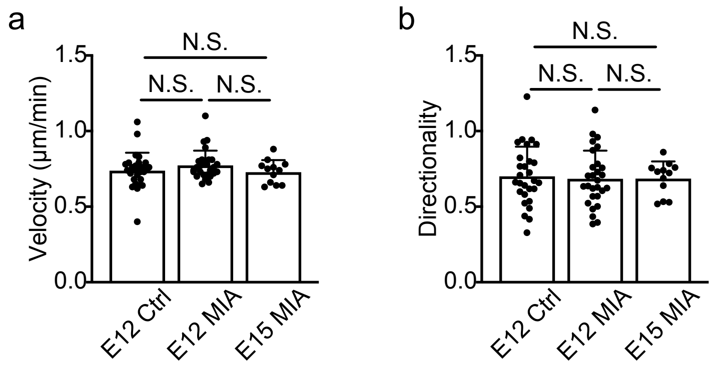

Averaged data for microglial tip velocity **(a)** and directionality **(b)** imaged *in vivo* from P42 mice offspring following injection of saline at E12 (E12 Ctrl) or Poly(I:C) at E12 (E12 MIA) or E15 (E15 MIA). Imaging was done under basal conditions (i.e. without LPS, cf. Figure 3 in main text). E12 saline and Poly(I:C) -injected mice; *n* = 28 cells from 8 mice in each group; E15 Poly(I:C) -injected mice; *n* = 12 cells from 4 mice.
N.S.: not significant, one-way ANOVA.

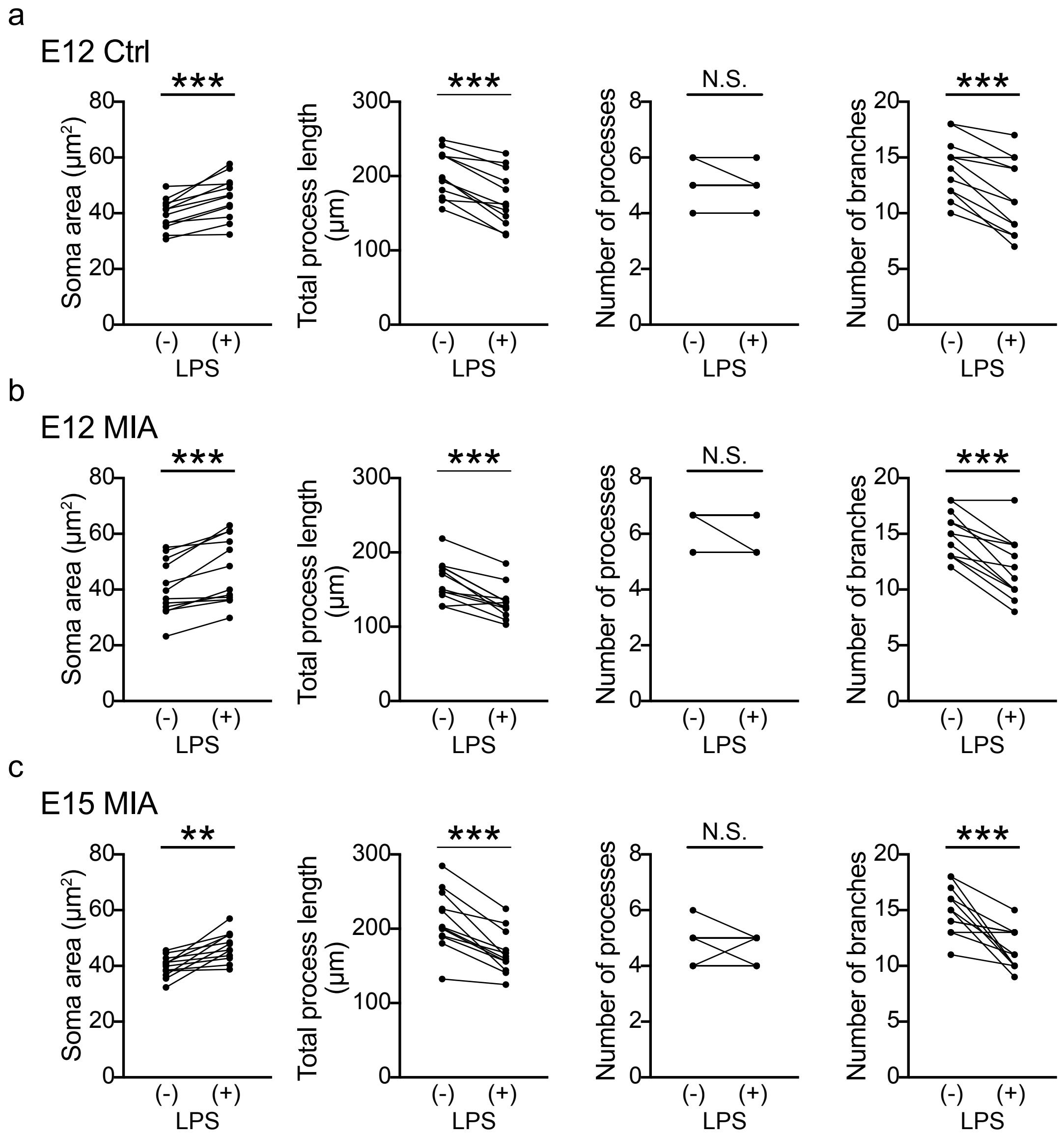

Effects of Lipopolysacharide (LPS) on microglial morphological parameters in P42 mice brains, previously injected with saline (Ctrl) or Poly (I:C) (MIA) at E12 or E15. In each panel, data comes from 12 cells from 4 mice in each group, with microglia located 100–200 μm depth below the brain surface.

Each point represents data from a single cell, with lines connecting data before and after LPS in that cell. $^{**}P < 0.01$, $^{***}P < 0.001$ and N.S.: not significant, unpaired *t*-test.
