# Supplementary material for: Maternal immune activation induces sustained changes in fetal microglia motility
Source: Sci Rep. 2020 Dec 7;10:21378. doi: 10.1038/s41598-020-78294-2 (PMC7721716; doi:10.1038/s41598-020-78294-2)
Supplement: Supplementary file 2 — Supplementary Figure S2. [file 41598_2020_78294_MOESM2_ESM.pdf]

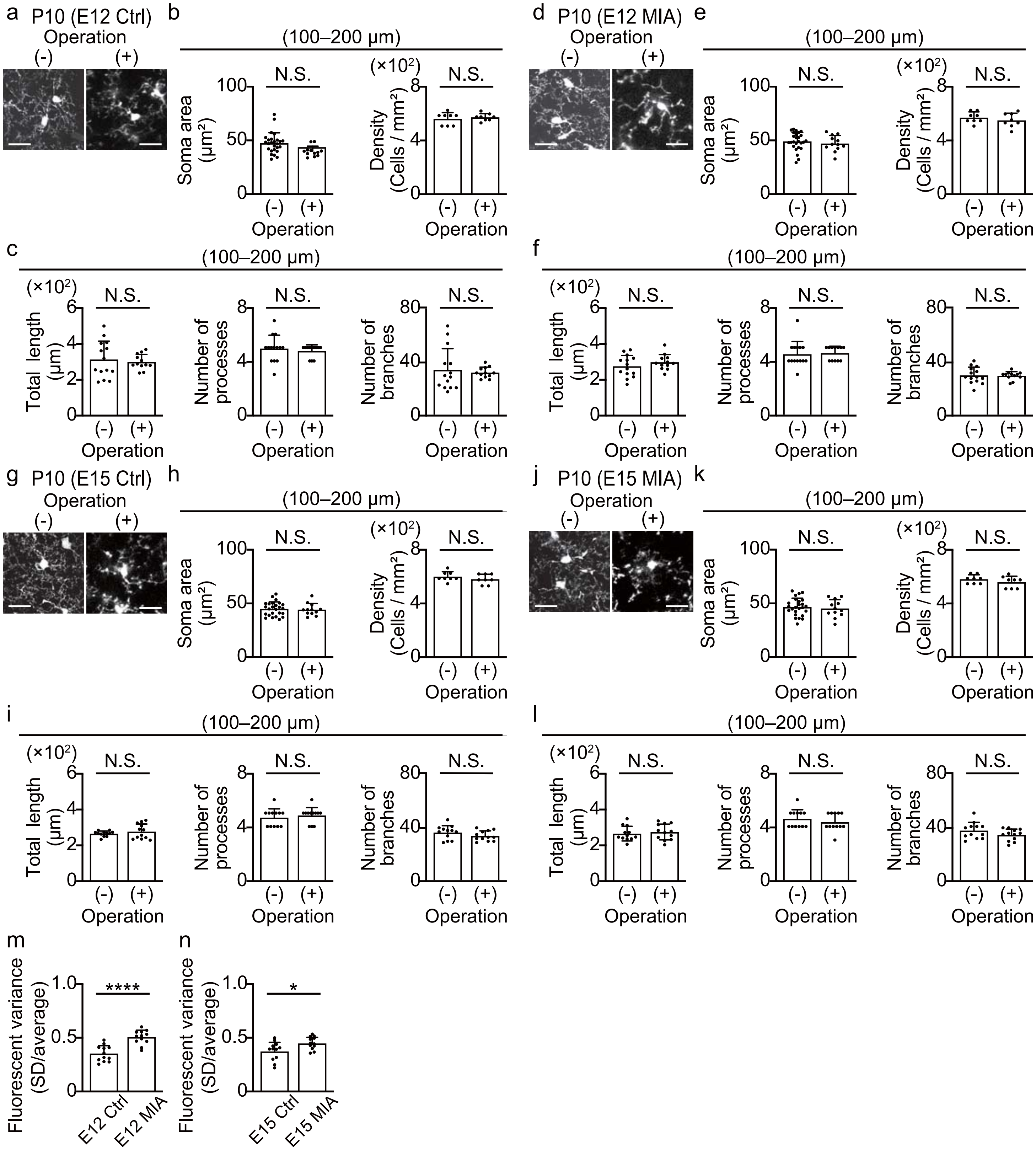

Quantification of microglial morphology at P10 following prior saline (Ctrl) or Poly (I:C) (MIA) at E12 or E15 as indicated. **(a-l)** Images for analysis were obtained both *in vivo*, using two-photon imaging after cranial window surgery (operation (+)) and from *in vitro* fixed tissue using immunohistochemistry (operation (-)). Neither the craniotomy operation, nor the MIA had any effect on morphology in these P10 brains. **(m, n)** Averaged data for variance of primary process intensity at P10 microglia. Panels **(a, d, g, j)** show typical images with higher-magnification insets (Scale bars, 20 µm). Graphs in panels **(b, c, e, f, h, i, k, l, m, n)** show mean and SD of different morphological parameters, dots show data from single samples (a single microglia or a single field for the analysis of density). *$P < 0.05$, ***$P < 0.0001$, N.S.: not significantly different, unpaired *t*-test.
