# Supplementary material for: Maternal immune activation induces sustained changes in fetal microglia motility
Source: Sci Rep. 2020 Dec 7;10:21378. doi: 10.1038/s41598-020-78294-2 (PMC7721716; doi:10.1038/s41598-020-78294-2)
Supplement: Supplementary file 3 — Supplementary Figure S3. [file 41598_2020_78294_MOESM3_ESM.pdf]

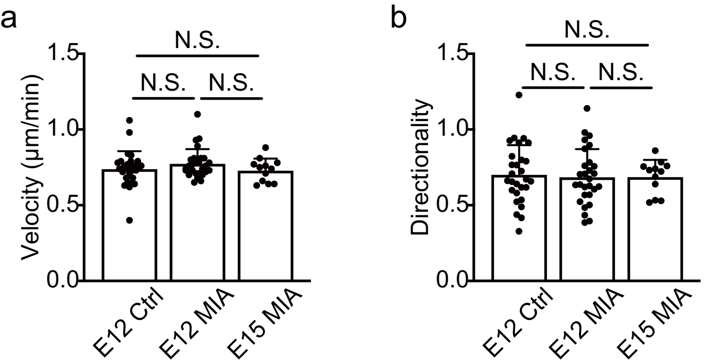

Averaged data for microglial tip velocity **(a)** and directionality **(b)** imaged *in vivo* from P42 mice offspring following injection of saline at E12 (E12 Ctrl) or Poly(I:C) at E12 (E12 MIA) or E15 (E15 MIA). Imaging was done under basal conditions (i.e. without LPS, cf. Figure 3 in main text). E12 saline and Poly(I:C) -injected mice; *n* = 28 cells from 8 mice in each group; E15 Poly(I:C) -injected mice; *n* = 12 cells from 4 mice. N.S.: not significant, one-way ANOVA.
