# Supplementary material for: Maternal immune activation induces sustained changes in fetal microglia motility
Source: Sci Rep. 2020 Dec 7;10:21378. doi: 10.1038/s41598-020-78294-2 (PMC7721716; doi:10.1038/s41598-020-78294-2)
Supplement: Supplementary file 4 — Supplementary Figure S4. [file 41598_2020_78294_MOESM4_ESM.pdf]

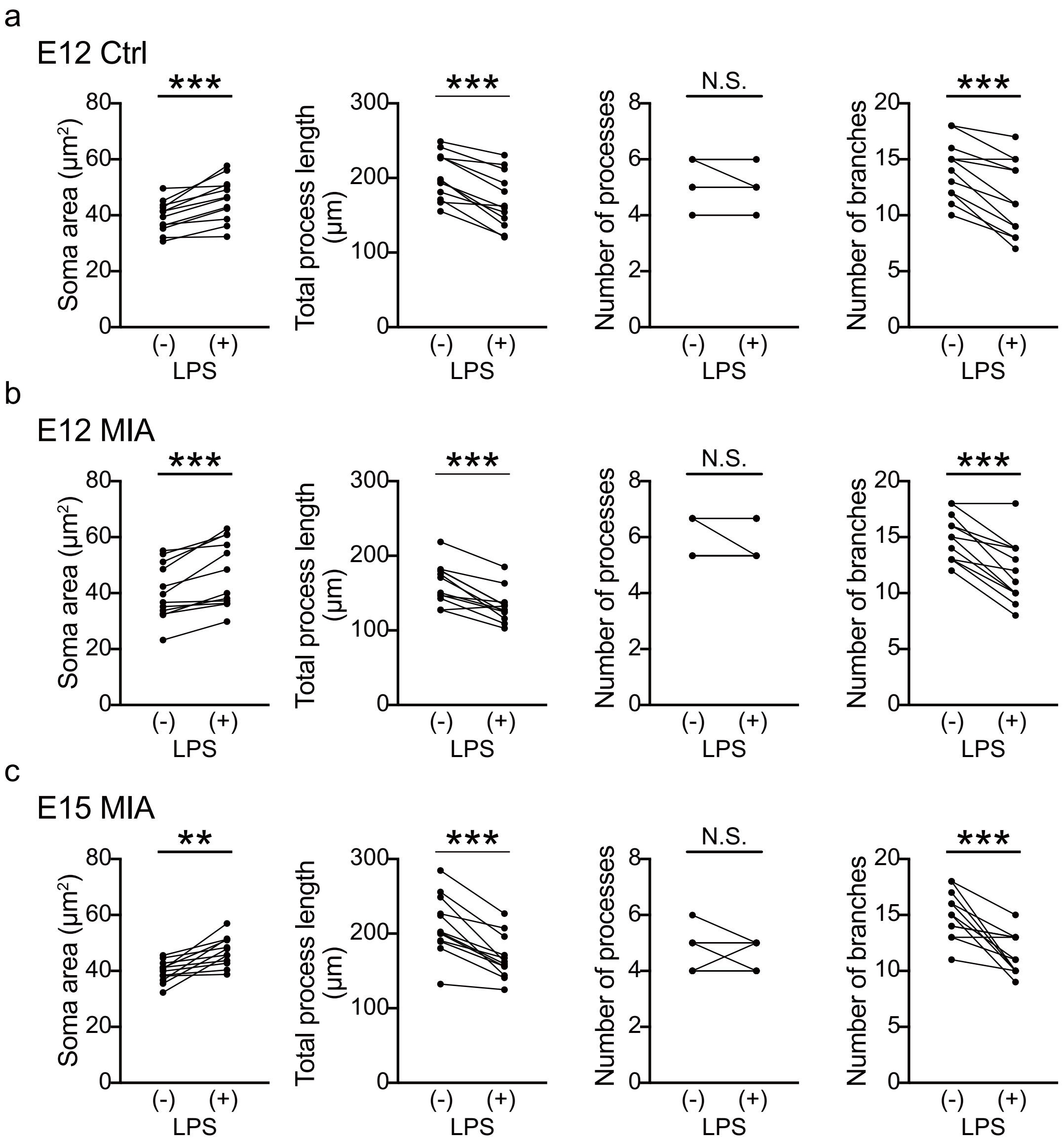

Effects of Lipopolysacharide (LPS) on microglial morphological parameters in P42 mice brains, previously injected with saline (Ctrl) or Poly (I:C) (MIA) at E12 or E15. In each panel, data comes from 12 cells from 4 mice in each group, with microglia located 100–200 µm depth below the brain surface.

Each point represents data from a single cell, with lines connecting data before and after LPS in that cell. ***P* < 0.01, ****P* < 0.001 and N.S.: not significant, unpaired *t*-test.
